# Genetic control of generative cell shape by DUO1 in *Arabidopsis*

**DOI:** 10.1007/s00497-023-00462-x

**Published:** 2023-04-06

**Authors:** Abdur Rauf, Hoda Khatab, Michael Borg, David Twell

**Affiliations:** 1 Department of Genetics and Genome Biology, University of Leicester, Leicester, LE1 7RH UK; 2 Department of Botany, Garden Campus, Abdul Wali Khan University Mardan, Mardan, Pakistan; 3 Department of Botany, Faculty of Science, University of Omar Al-Mukhtar, Al-Baida, Libya; 4 Department of Algal Development and Evolution, Max Planck Institute for Biology Tübingen, Max-Planck-Ring 5, 72076 Tübingen, Germany

**Keywords:** Arabidopsis, Pollen, Generative cell, Sperm cell, Morphogenesis

## Abstract

**Key message:**

The main features of generative cell morphogenesis, formation of a cytoplasmic projection and elongation of the GC body, operate through independent genetic pathways.

**Abstract:**

Male gametogenesis in developing angiosperm pollen involves distinctive changes in cell morphogenesis. Re-shaping and elongation of the generative cell (GC) are linked to the formation of a GC cytoplasmic projection connected to the vegetative cell nucleus. Although genetic control of GC morphogenesis is unknown, we suspected the involvement of the germline-specific MYB transcription factor DUO POLLEN1 (DUO1). We used light and fluorescence microscopy to examine male germline development in pollen of wild-type Arabidopsis and in four allelic *duo1* mutants expressing introduced cell markers. Our analysis shows that the undivided GC in *duo1* pollen forms a cytoplasmic projection, but the cell body fails to elongate. In contrast GCs of cyclin-dependent kinase function mutants, which fail to divide like *duo1* mutants, achieve normal morphogenesis. We conclude that DUO1 has an essential role in the elongation of the GC, but DUO1-independent pathways control the development of the GC cytoplasmic projection. The two main features of GC morphogenesis therefore operate through independently regulated genetic pathways.

**Supplementary Information:**

The online version contains supplementary material available at 10.1007/s00497-023-00462-x.

## Introduction

Plant fertility depends upon de novo generation of a separate germ cell lineage in developing pollen grains. In angiosperms, this process involves asymmetric mitotic division of a unicellular microspore to form a larger vegetative cell (VC) and a smaller generative cell (GC). The GC is first engulfed by the VC to form a cell-within-a-cell and then divides to form the pair of sperm cells (SCs) required for double fertilisation. This unique compartmentalisation of the germline enables the male gametes to be delivered to the ovules by the growing pollen tube (Borg and Twell 2011; Berger and Twell 2011; Twell 2011).

Generative cells of angiosperms are known to undergo distinctive changes in morphogenesis based on studies of a wide range of species (Sanger and Jackson 1971; Palevitz and Tiezzi 1992; Yu and Russell 1992, 1994; Russell et al. 1996; Russell and Strout 2005; Eva et al. 2006; Oh et al. 2010). These include studies of tricellular and bicellular pollen species, where GC division takes place before or after pollen germination respectively and a general scheme is recognised (Tables S1 and S2). Immediately after asymmetric division of the microspore, the newly formed GC is lenticular (lens-shaped) when attached to the pollen wall but becomes spheroidal after it is engulfed as a separate body within the VC cytoplasm. Subsequently, the GC elongates to become fusiform (spindle-like) or lachrymiform (elongated drop-shaped) before dividing to form the sperm cell pair.

Generative cell morphology is described mainly from ultrastructural studies and from immunofluorescence labelling of microtubules (MTs) in fixed samples (Sanger and Jackson 1971; Palevitz and Cresti 1989; Palevitz 1993; Zhou and Yang 1991; Russell et al. 1996). This has allowed detailed 3-D reconstruction such as in *Rhododendron*, which has an elongated GC body of 17 × 3.5 µm length and width extending over 100 µm when coiled cytoplasmic projections (also known as extensions or ‘tails’) of the GC are included (Theunis et al. 1985). Studies involving immunolabelling of MTs have revealed further details and an exceptionally elongated GC of around 250 µm in pollen tubes of *Gagea lutea* (Zhang et al. 1995).

The GC and subsequently the SCs are physically linked to the vegetative cell nucleus (VN) via one or more cytoplasmic projections to form a structural assemblage known as the male germ unit (MGU) (Dumas et al. 1985; reviewed in Mogensen 1992; McCue et al. 2011). Ultrastructural studies show that the GC may have a single projection as in cotton (Jensen et al. 1968), a projection at both ends as in *Rhododendron* (Theunis et al. 1985), or a variable number between one and four as in *Cymbidium* (Yu and Russell 1992). The cytoplasmic projections of the GC and/or SC have also been visualised with fluorescent marker proteins in live cells of tobacco (Oh et al. 2010), maize (Kliwer and Dresselhaus 2010) and Arabidopsis (Ge et al. 2011; Boavida et al. 2013).

Elongated GCs and SCs typically possess a prominent longitudinal array of bundled MTs surrounding the nucleus (reviewed in Palevitz and Tiezzi 1992), extending at one or both ends into a cytoplasmic projection (Palevitz and Cresti 1988; Del Casino et al. 1992; Zee and Ye 2000; Oh et al. 2010; Kliwer and Dresselhaus 2010). This specialised MT system is thought to play a structural role in re-shaping cells of the male germ lineage and maintaining gamete morphology (Sanger and Jackson 1971; Palevitz 1993; Zhang et al. 1995; Russell and Strout 2005). For example, destruction of the GC MT array with anti-MT-drugs results in reversion of GCs to a spheroidal shape (Sanger and Jackson 1971). Moreover, isolated GCs or SCs become spheroidal after isolation and the MT cage is lost (Tanaka 1988; Theunis et al. 1992).

Here, we investigated GC morphogenesis and its genetic control by known regulatory proteins. Previous studies of Arabidopsis GC development have used fixed and sectioned material (Owen and Makaroff 1995; Kuang and Musgrave 1996) and fluorescent labelling of nuclei (Park et al. 1998; Lalanne and Twell 2002). We used a panel of fluorescent protein markers to study GC morphogenesis in wild-type and mutant lines. The markers label either the plasma membrane (Boavida et al. 2013) or the nuclear membrane (Rose and Meier 2001), VC and GC nuclei (Brownfield et al. 2009b) or MTs in male germ cells (see materials and methods). We describe two main phases of GC morphogenesis involving formation of the GC cytoplasmic projection and elongation of the GC body.

Our hypothesis was that known regulators of male germline development might also play a role in GC morphogenesis, and we focussed on the MYB transcription factor DUO1 POLLEN1 (DUO1), which regulates both the division of the GC and the differentiation of sperm cells (Rotman et al. 2005; Brownfield et al. 2009a; Borg et al. 2011). We also examined GC morphogenesis in CDKA;1-function mutants that block GC division in pollen, but which do not prevent gamete differentiation (Nowack et al. 2006; Kim et al. 2008; Brownfield et al. 2009a). This approach enabled us to distinguish regulatory proteins with roles restricted to cell cycle progression from those involved in discrete aspects of male germ cell morphogenesis. Our results demonstrate an essential role for DUO1 in GC elongation that is independent of the morphogenetic pathways required to establish the GC projection.

## Materials and methods

### Plant material and growth conditions

*Arabidopsis thaliana* (L.) Heynh. plants were grown on a 1:1 mixture of Levington F2S compost (Scotts, UK) and vermiculite at 22 ℃ with a 16-h-light and 8-h-dark cycle or with 24 h light (120 to 140 µ mol/m^2^/s) with 50–60% relative humidity. Details of the mutant alleles of genes that result in the formation of bicellular pollen are given in Table S5. Four mutant alleles of *DUO1* were examined: *duo1-1* (Durbarry et al. 2005; Rotman et al. 2005), *duo1-2* (Rotman et al. 2005), *duo1-3* (this study) and *duo1-4* (Borg et al. 2014). Seeds of the transposon insertion line GT_5_18345 in At3g60460 were obtained from the Nottingham Arabidopsis Stock Centre (NASC) and renamed *duo1-3*. Single mutant alleles were examined for *fbl17* (Kim et al. 2008) and *cdc2a-1* (Nowack et al. 2006), which is referred to as *cdka;1* (Iwakawa et al. 2006) in the main text.

A range of previously described markers and transgenic lines were used (Table S3). Seeds of the TET11-GFP marker were a kind gift of Leonor Boavida, where TET11 is a member of the plasma membrane integral protein family, TETRASPANIN (Boavida et al. 2013). The DUO3:H2B-GFP marker labels both VC and GC nuclei (Brownfield et al. 2009b). The proHTR10:GFP-TUA6 marker was built by replacing the *NTM19* promoter from ProNTM19:GFP-TUA6 (Oh et al. 2010) with the *HTR10* promoter. The DUO1:TET11-tdTomato/GFP, MBD10:TET11-tdTomato/GFP, LAT52:RanGAP-GFP/tdTomato and DUO3:H2B-tdTomato markers were produced by combining entry clones using MultiSite Gateway® technology (Invitrogen) as described (Brownfield et al. 2009a; Borg et al. 2011) (Table S3). Homozygous single- and double-fluorescent marker lines were generated in wild-type *A. thaliana* and in mutant backgrounds either by crossing or by *Agrobacterium tumefaciens* (GV3101) mediated transformation using standard methods (Table S4). Transformants were selected on soil supplemented with 30 µg/ml BASTA (glufosinate ammonium; DHAI PROCIDA) fed by subirrigation or on Murashige and Skoog agar containing 50 µg/ml kanamycin or 20 µg/ml hygromycin and grown to maturity as described above.

### Microscopy

To examine developing pollen for bright field analysis and for GFP and RFP marker expression, anthers from each of the four long (medial) stamens of successive flower bud stages were dissected into 5–10 µl of 0.3 M mannitol on a glass slide using a 25-gauge hypodermic needle. Flower developmental stages were staged as described (Lalanne and Twell 2002); + 1, first open flower (mature tricellular pollen), − 1 and − 2, first and second unopened flower buds (immature tricellular pollen), − 3, third unopened bud (pollen mitosis II and bicellular pollen), − 4, fourth unopened bud (bicellular pollen). Pollen was mounted in 0.3 M mannitol and examined using a Nikon Eclipse-80i microscope. Bright field and differential interference contrast (DIC) images were captured with a Nikon-D100 (Model MH-18, Japan) and fluorescence images were captured with a HAMAMATSU ORCA-ER (Model C4742-95, Japan) using NIS-Elements software. For examination of pollen nuclei labelled with DAPI (4′-6-Diamidino-2-phenylindole dihydrochloride), pollen was incubated in 0.8 µg/ml DAPI as previously described (Brownfield et al. 2009a). GC dimensions including major axis, minor axis, aspect ratio and circularity were determined using ImageJ (http://rsb.info.nih.gov/ij/).

For confocal microscopy (CLSM) developing pollen mounted in 0.3 M mannitol was examined with an Olympus-FV1000 (USA) microscope and Fluoview viewer software (FV10-ASW Version 04.00.02.09). Optical stacks were taken with an immersion-oil UPLSAPO 60x/1.35NA objective. GFP signal was imaged with 488 nm excitation and 500–600 nm detection. RFP was excited at 635 nm with detection of 655–755 nm. Hoechst signal was imaged with 405 nm excitation and an emission of 425–475 nm. Images were taken using the same voxel (volume pixel) resolution (*XY* dimension 85 nm, *Z* dimension 250 nm). CLSM images were transformed into 3-D reconstructions with IMARIS software (Bitplane AG, An Oxford Instruments Company Badenerstrasse, Zurich, Switzerland).

## Results

### Analysis of cellular morphogenesis in wild-type pollen

**Fig. 1:**
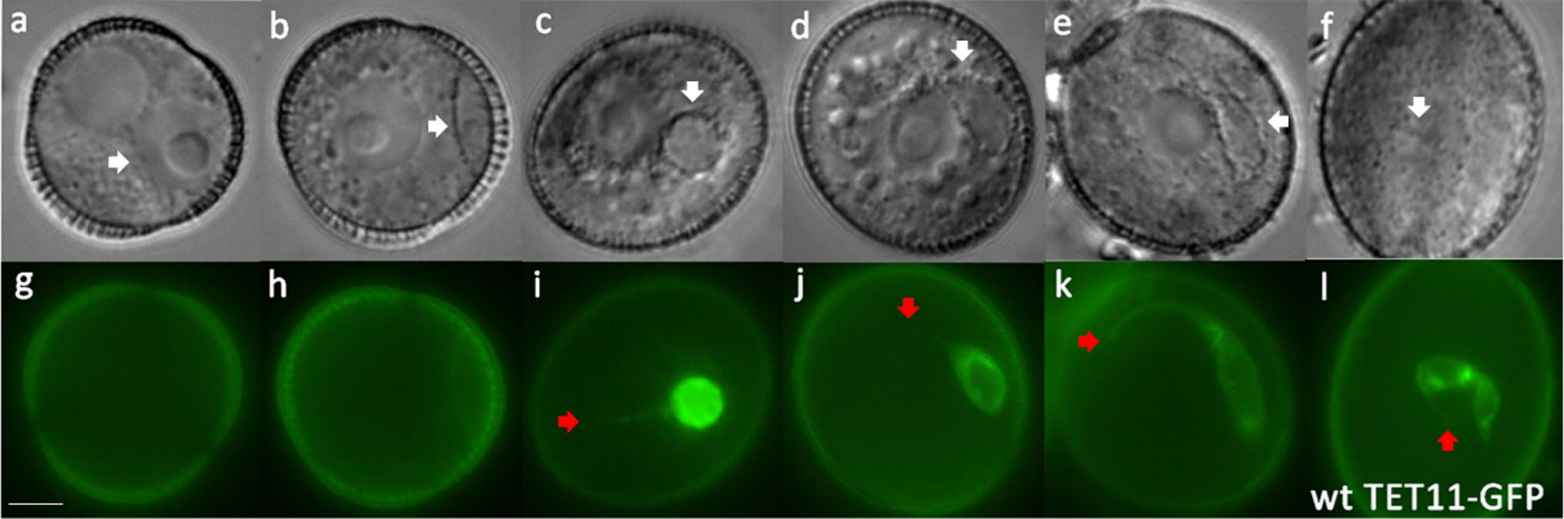
Generative cell morphogenesis in wild-type Arabidopsis pollen. Corresponding DIC (**a**–**f**) and fluorescence (**g**–**l**) images of progressive stages of developing pollen expressing the germline plasma membrane marker TET11:GFP. **a** Polarised microspore, **b** GC attached to pollen wall, **c** round GC, **d** partially elongated GC, **e** fully elongated GC, **f** early tricellular pollen. The generative cell (GC) and pair of sperm cells (SC) are indicated with white arrows, while the fine cytoplasmic projection of the GC or SC is indicated with a red arrow. Representative images are shown of eight plants examined. Scale bar = 5 µm

**Fig. 2:**
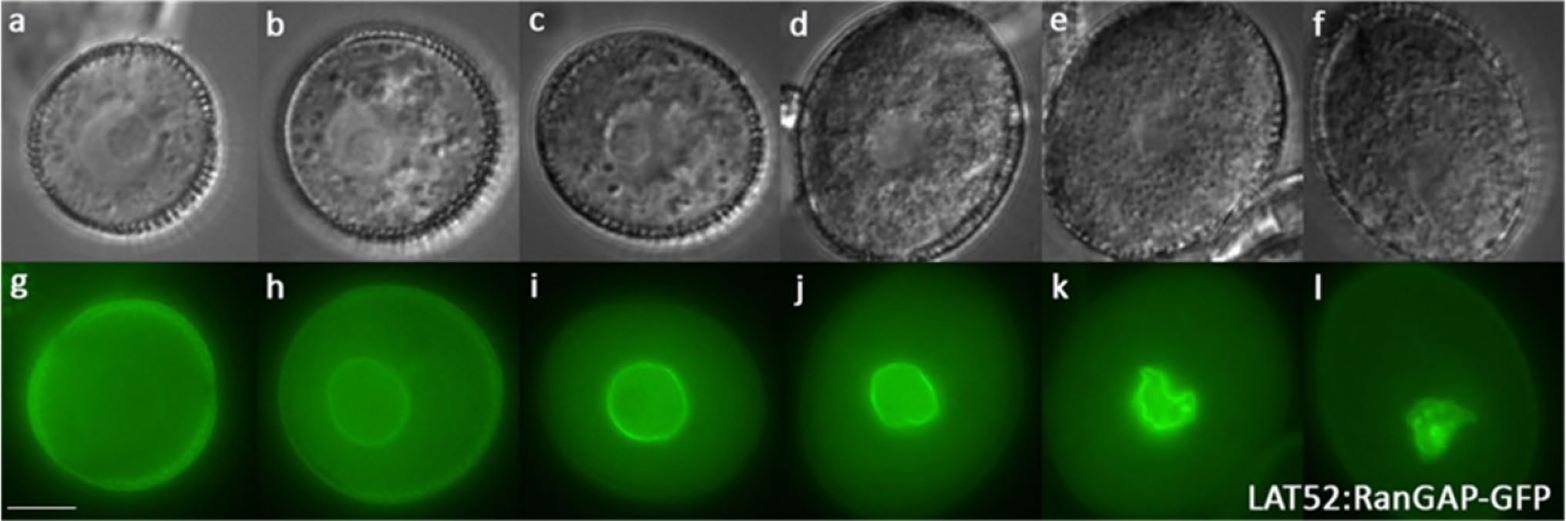
Morphogenesis of the vegetative cell nucleus in developing Arabidopsis pollen. Corresponding DIC (**a**–**f**) and fluorescence (**g**–**l**) images of developing pollen expressing the VC nuclear envelope marker LAT52:RanGAP-GFP marker. The VC nuclear membrane becomes highly lobed in tricellular and mature pollen stages (**k**, **l**). **a**, **g** Unpolarised microspore, **b**, **h** early bicellular, **c**, **i** mid-bicellular, **d**, **j** late bicellular, **e**, **k** late tricellular and **f**, **l** mature pollen. Representative images are shown of 11 plants examined. Scale bar = 5 µm

We first examined cellular morphogenesis in developing Arabidopsis pollen using differential interference contrast (DIC) and fluorescence microscopy. DIC was used to monitor GC development at bicellular pollen stages, while introduced transgenic cell markers allowed GC cytoplasmic projections as well as sperm cell morphology to be visualised in tricellular pollen stages. The cell markers used and their expression patterns are summarised in tables S3 and S4. In wild-type pollen, TET11-GFP and HTR10:GFP-TUA6 mark the plasma membrane and cytoplasm of male germline cells respectively (Figs. 1, S1; 3, S3), while DUO3:H2B-tdTomato marks both germline and vegetative nuclei (Figs. S2, S4). LAT52:RanGAP-RFP marks only vegetative cell nuclear membrane (Fig. 2). Further, the markers driven by the TET11 and DUO3 promoters are detectable throughout bicellular and tricellular pollen stages (Figs. S1, S2, S4). Observations made with the above-mentioned markers resolved GC morphogenesis into sequential phases; the first wherein the GC projection is initiated followed by a second, where elongation of the main body of the GC occurs.

### Male germline morphogenesis

**Fig. 3:**
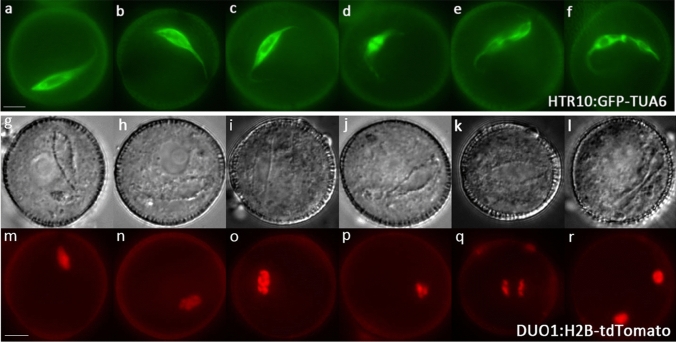
Arabidopsis male germline morphogenesis at late bicellular to early tricellular pollen stages. **a**–**f** Progressive stages of late bicellular (**a**–**c**) or early tricellular (**d**–**f**) pollen labelled with the cytoplasmic germline marker HTR10:GFP-TUA6. Elongated GCs prior to and during division (**a**–**d**), and newly formed sperm cells (**e**–**f**), possess one (**a**, **b**) or two (**c**–**f**) cytoplasmic projections. Cytoplasmic projections persist during anaphase (**d**). **g**–**r** Corresponding DIC (**g**–**l**) and fluorescence (**m**–**r**) images of pollen labelled with the nuclear germline marker DUO1:H2B-tdTomato at progressive stages of GC mitosis. The elongated shape of the GC persists throughout mitosis, from prophase (**g**, **m**; **h**, **n**; **i**, **o**), through metaphase (**j**, **p**), anaphase (**k**, **q**) and telophase (**l**, **r**). Representative images are shown of 9 (HTR10:GFP-TUA6) or 7 (DUO1:H2B-tdTomato) plants examined. Scale bar = 5 µm

In common with most angiosperms (Tables S1 and S2), Arabidopsis GCs undergo elongation prior to division at pollen mitosis II (Fig. 1b–e, h–k). The newly formed lenticular GC is attached to the pollen wall immediately after PMI (Fig. 1b, h). The internal wall separating the GC from the VC becomes domed and rounds up before detachment from the pollen wall (Figs. 1b, c, i; S1). At early bicellular pollen stage, the fully detached GC is spheroidal and positioned close to the centrally located VN, which has a large nucleolus and round profile (Figs. 1c, i, 2b, h; Movie 1). Lipid droplets accumulate around the plasma membrane of the GC and its cytoplasmic projection, which provides a useful marker for GC morphology with DIC microscopy (Fig. 1b–e). The GC projection is formed prior to elongation of the GC body (Figs. 1c; S3; Movie 2). By mid-bicellular stage, the GC undergoes polar elongation to form a tear drop shape with its axis aligned with the cytoplasmic projection (Fig. 1d; Movies 3–4). The GC body and nucleus both elongate and maximum length occurs just before GC division at PMII (Fig. 1e). Between early bicellular stage (when the nucleus is spheroidal) to late bicellular stage, the mean aspect ratio increases from 1.02 to 3.07 due to an increase in the mean major axis from 4.29 to 10.29 µm and a reduction in the mean minor axis from 4.87 to 3.3 µm (*P* < 0.05, Fig. 6; Table S6). While most GCs possess a single cytoplasmic projection (95.1%) axially aligned with the elongated body of the GC (Fig. 3a–c; Table S7: Movie 5), the remainder possess an extension at either end, typically of unequal length (Movie 6).

### Sperm cell morphogenesis before anthesis

The GC is associated with the VN before it divides to form two elongated SCs that remain tightly associated with the VN at late tricellular stage (Figs. 1e/k, f/l, 4d/I, e/j). While the main bodies of each SC pair are similar in length, one SC typically has a long cytoplasmic projection, while the second SC lacks or has a shorter projection (Figs. 5c–e; 7d; Movies 7–8; Table S7). The proportion of sperm cell pairs with a single cytoplasmic projection (87%) was slightly lower than that of GCs with a single projection (95.1%, Table S7), but these values did not differ significantly (*P* = 0.06), indicating limited development of a second projection after division of the GC. The length of the cytoplasmic projection is often equal to or greater than that of the SC body and SCs remain connected through a cytoplasmic bridge with a knot-like structure (Fig. 5d; Movies 8–9) as described by Boavida et al. (2013). It is also notable that SCs initially possess nuclei which are round in profile, but these elongate to an elliptical shape with pollen maturity (Fig. 7b–d).

**Fig. 4:**
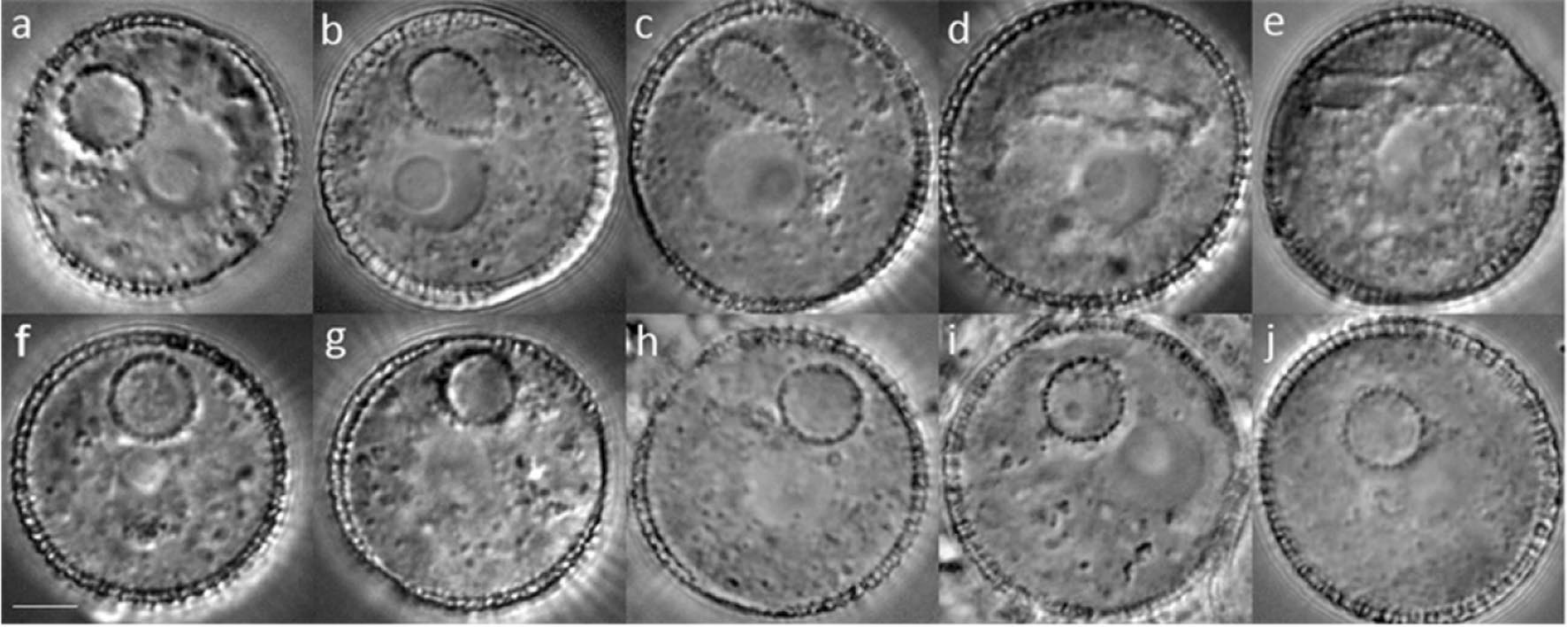
Abnormal GC morphogenesis in *duo1-4* pollen. Representative DIC images of pollen from heterozygous *duo1-4*^±^ plants at progressive bud stages showing normal (**a**–**e**) and abnormal (**g**–**j**) GC morphology. At early bicellular stage **a**, **f** the GC appears uniformly round in the pollen population. **b**, **c** Partially elongated GC, **d** fully elongated GC and **e** sperm cell pair in early tricellular pollen. Segregating abnormal (*duo1-4*) pollen in which the undivided GC fails to elongate (**g**–**j**). Scale bar = 5 µm

**Fig. 5:**
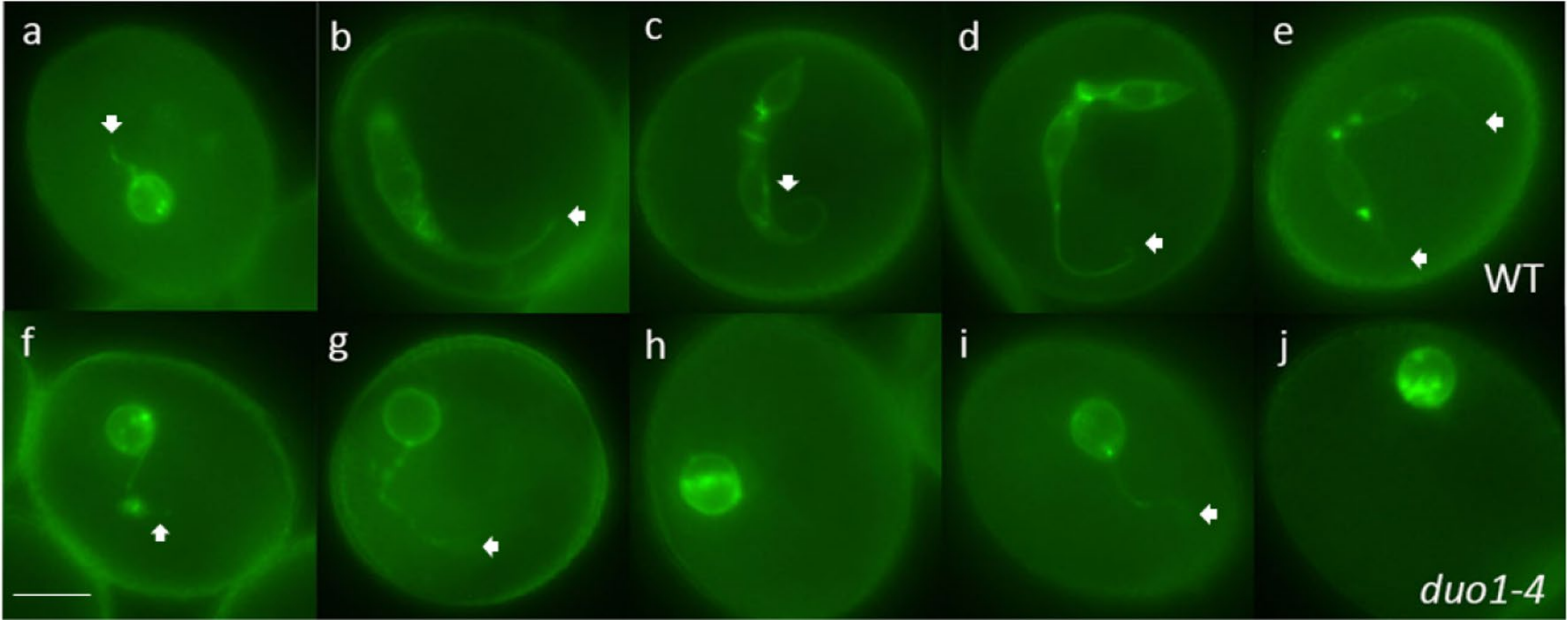
Undivided *duo1-4* generative cells form a cytoplasmic projection but fail to elongate. Corresponding fluorescence images of segregating normal (**a**–**e**) wild-type (WT) and abnormal (**g**–**j**) mutant (*duo1-4*) pollen from heterozygous *duo1-4*^±^ plants marked with the germline plasma membrane marker TET11:GFP. At early bicellular stage (**a**, **f**) the GC appears uniformly round in the pollen population with a clear cytoplasmic projection. Normal GCs elongate (**b**) and divide to form two elongated sperm cells (**c**–**e**) with clear a cytoplasmic projection, while abnormal (*duo1-4*) GCs (**g**–**j**) fail to elongate but still possess a cytoplasmic projection (white arrows). Normal and abnormal pollen are taken from the same bud at each stage. Scale bar = 5 µm

### Morphogenesis of the vegetative cell and nucleus

The VC nucleus in Arabidopsis pollen undergoes morphological changes similar to those observed in other angiosperms (Mogensen 1986; McConchie et al. 1985; Hu and Yu 1988; Eva et al. 2006). The VC nucleus has a prominent nucleolus and is surrounded by a smooth nuclear envelope with a round profile at early to mid-bicellular pollen stages (Fig. 2h–i). By late bicellular stage, the VC nucleus no longer has a smooth outline and by late tricellular bud stages is highly lobed or folded (Fig. 2k, l: Movie 10–11). This re-shaping of the VC nucleus from spherical to lobed is clearly observed with different cellular markers (Fig. S4b–n; Movie 10–11).

### The elongated GC maintains a cytoplasmic projection during pollen mitosis II

Dynamic changes in GC morphogenesis involving loosening of the close physical association between the GC and VC nucleus and partial or complete loss of the GC projection have been reported in tobacco (Palevitz 1993; Yu and Russell 1994). In Arabidopsis pollen labelled with HTR10:GFP-TUA6, we observed that the cytoplasmic projection and the elongated shape of the GC (Fig. 3a–c) were maintained during anaphase of GC mitosis (Fig. 3d). We also examined GC morphology at progressive stages of GC mitosis in pollen labelled with the nuclear germline marker DUO1:H2B-tdTomato (Fig. 3g–r). These observations clearly show that the GC elongation is maintained throughout all stages of mitosis (Fig. 3g–l; m–r; Movies 5–7).

### GC morphogenesis is DUO1-dependent

We examined GC morphogenesis in developing pollen released from buds of heterozygous *duo1-4* mutants using DIC optics. Pollen was scored as bicellular when the GC was round (RD), semi-elongated (ED1) or elongated (ED2), or tricellular pollen (TCP) when two sperm cells were present. While half of the pollen population of heterozygous *duo1-4* mutants completed GC elongation and division to form two sperm cells (Fig. 4a–e), GCs in the remaining half failed to elongate (Fig. 4f–j; S6a). Similarly, the analysis of heterozygous mutants for each of three independent *duo1* alleles (*duo1-1*, *duo1-2, duo1-3*) at late bicellular/early tricellular stages using DIC microscopy showed that GCs lacked elongation for approximately half of the pollen population (Fig. S6b–d).

To distinguish the identity of segregating WT and *duo1-4* pollen, we created a *duo1-4*^±^ double nuclear marker line, homozygous for DUO1:H2B-tdTomato and HTR10:H2B-GFP. While all pollen grains of the WT double marker line express RFP and GFP, *duo1-4*^±^ segregates 100% RFP and 50% GFP due to lack of HTR10:H2B-GFP expression in *duo1-4* pollen. This analysis confirmed failure of the GC and GC nucleus to elongate in *duo1-4* pollen (Fig. S7).

**Fig. 6:**
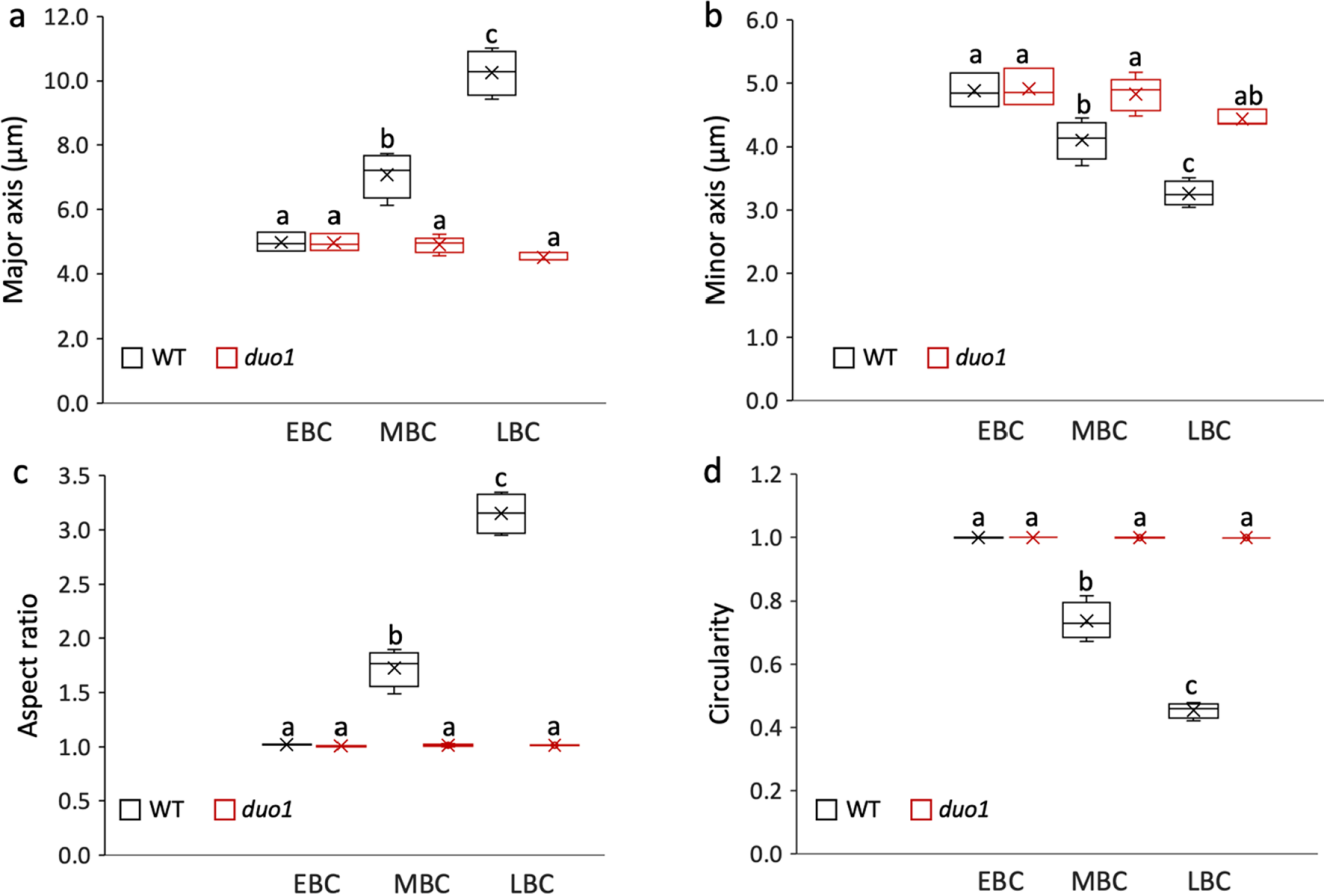
Developmental analysis of GC shape. The dimensions of GCs at three stages of development (EBC, MBC and LBC) were analysed from WT pollen and from *duo1* pollen segregating in homozygous TET11-GFP/HTR10:H2B-RFP marker plants. Box plots show the distribution of GC dimension parameters for major axis (**a**), minor axis (**b**), aspect ratio (**c**) and circularity (**d**) calculated in ImageJ. Centre lines show medians and crosses means in the boxed interquartile range; whiskers extend 1.5 times the interquartile range. Letters above whiskers indicate significantly different means (*P* < .05) determined with One-Way ANOVA and Tukey’s HSD Test for multiple comparisons. EBC, early bicellular pollen; MBC, mid-bicellular pollen; LBC; late bicellular pollen

The dimensions of GCs were determined at three stages of development (EBC, MBC and LBC) from WT pollen and from *duo1* pollen segregating in homozygous TET11-GFP/HTR10:H2B-RFP marker plants. While there was an approximately threefold increase in the mean aspect ratio of WT GCs during development mainly due to an increase in increase in length of the major axis, mutant GCs in *duo1* pollen did not show a significant change in aspect ratio between EBC and LBC stage pollen (Fig. 6a–c; Table S6). Further, the calculated circularity of *duo1* GCs remained close to that of a perfect circle (1.0) throughout development, while WT GCs showed progressive and significant deviations at EBC and LBC stages (Fig. 6d).

Independent analysis of TET11-GFP marked *duo1-4*^±^ plants revealed that mutant GCs fail to elongate but can still form a long cytoplasmic projection (Fig. 5f–j; Movie 12). Similarly, the analysis of three further TET11-GFP marked mutant alleles of *DUO1* (*duo1-1*, *duo1-2, duo1-3*) confirmed failure of GC body elongation (Fig. S5e–h), but retention of the ability of mutant GCs to form a GC projection. Scoring of the number of GC projections in *duo1-1* and *duo1-3* pollen revealed that over 90% of undivided mutant GCs formed single rather than double projections like the WT (Table S7), and the proportion of pollen with one or two projections did not differ significantly between WT and *duo1* alleles at GC stage (*P* = 0.51) or at SC stage (*P* = 0.29). Overall, these data demonstrate that DUO1 is required for elongation of the GC body but not for growth of the GC projection.

A limitation of the TET11-GFP plasma membrane marker is its inability to differentiate WT and mutant GCs at early or mid-bicellular bud stages prior to GC elongation. Therefore, double-labelled *duo1-4*^±^ TET11-GFP HTR10:H2B-RFP marker lines were created which lack nuclear RFP expression in *duo1-4* pollen. Analysis of homozygous marker lines revealed that all WT (GFP + RFP +) GCs showed normal morphogenesis (Figs. S8a–h, S9a–j), while mutant GCs (GFP + RFP –) failed to elongate (Figs. S8i–p, S9k–t), but were able to form a cytoplasmic projection (Fig. S8k). In conclusion, elongation of the GC body is strictly dependent on DUO1, whereas formation of the GC cytoplasmic projection is not.

A summary of the extended roles of DUO1 in Arabidopsis germline development is presented in Fig. 8. In the absence of DUO1 function, the GC fails to elongate but still forms a cytoplasmic projection. The uncoupling of these two processes indicates that DUO1 has a specific role in GC morphogenesis and that formation of the cytoplasmic projection and elongation of the GC body are independently regulated.

### GC morphogenesis is unaffected in CDKA function mutants

**Fig. 7:**
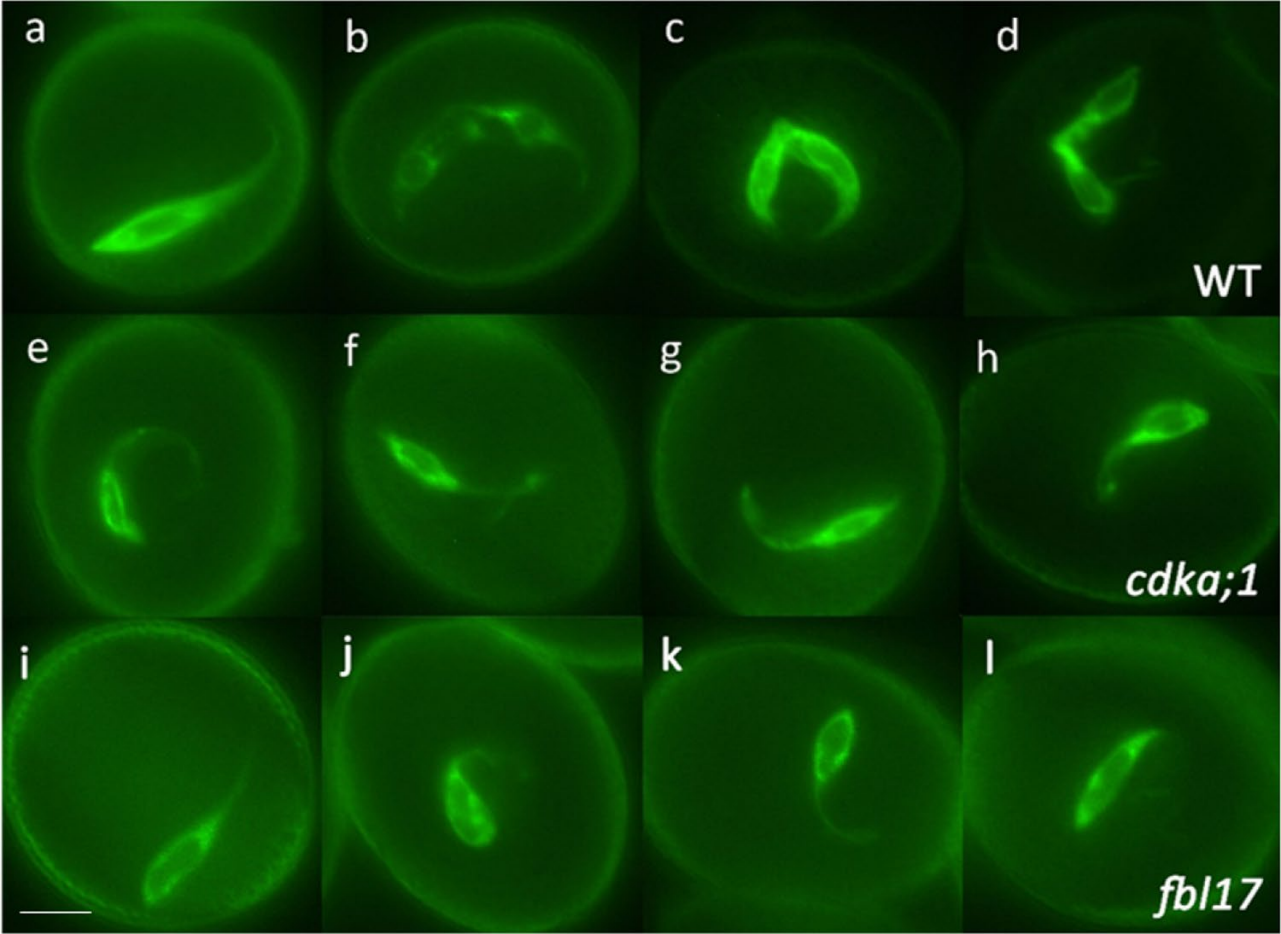
Normal GC morphogenesis in CDKA function mutants. Corresponding fluorescence images of wild-type (**a**–**d**), *cdka;1* (**e**–**h**) and *fbl17* (**i**–**l**) pollen at late bicellular (**a**, **e**, **i**), early tricellular (**b**, **f**, **j**), mid-tricellular (**c**, **g**, **k**) and late tricellular (**d**, **h**, **l**) pollen stages marked with HTR10:GFP-TUA6. Undivided mutant GCs in *cdka;1* and *fbl17* pollen elongate normally and possess a long cytoplasmic extension, like wild-type GCs in late bicellular pollen (**a**). Scale bar = 5 µm

To further examine the link between incomplete GC morphogenesis and failure of GC division observed in *duo1* mutants, we investigated whether mutations specific for cell cycle progression also affect GC morphogenesis. Loss-of-function mutations in *CDKA;1*, the major cell cycle cyclin-dependent kinase (CDK) or the F-box protein gene, *F-BOX-LIKE 17* (*FBL17*), which regulates CDKA;1 activity via destruction of CDK inhibitors, both cause failure of GC division in pollen but do not disturb germ cell fate (Nowack et al. 2006; Iwakawa et al. 2006; Kim et al. 2008; Gusti et al. 2009; Brownfield et al. 2009a). Therefore, we introduced the HTR10:GFP-TUA6 and TET11-GFP markers into heterozygous *cdka;1*^±^ and *fbl17*^±^ mutants. While the GC divided to form two SCs in the wild-type half of both pollen populations (Figs. 7a–d; S10–S11), the GC failed to divide in *cdka;1* and *fbl17* pollen as expected (Fig. 7e–h, i–l). For both markers, we observed that all mutant GCs elongated normally and developed a long cytoplasmic projection (Fig. 7e–l). These results demonstrate that neither CDKA;1 nor FBL17 are required for normal GC morphogenesis, and highlight the independent control of GC morphogenesis and GC division, such that completion of normal morphogenesis does not inevitably result in GC division.

## Discussion

We examined cell morphogenesis in the developing male germline of Arabidopsis pollen and the contribution of the MYB transcription factor DUO1 to this process. In vivo analysis of multiple loss-of-function alleles of *duo1* (Figs. 4, 5; S5–S9) demonstrate that DUO1 is indispensable for elongation of the GC body, but is not required for the GC to form a cytoplasmic projection (Figs. 4, 5, 7). We also investigated whether GC morphogenesis was affected in null alleles of CDKA;1-function mutants which like *duo1*, prevent division of the GC in pollen (Nowack et al. 2006; Iwakawa et al. 2006; Kim et al. 2008; Gusti et al. 2009). Neither formation of the GC cytoplasmic projection nor elongation of the GC body were affected in CDKA;1-function mutants (Fig. 7; S10–12), highlighting the specific requirement of DUO1 for GC body elongation. Our results show that formation of the GC cytoplasmic projection and axial elongation of the GC body are developmentally phased but independently regulated processes.

### Male germline morphogenesis in Arabidopsis and other angiosperms

The VC has a vital role in the orderly transport of germline cells within the pollen tube. The GC also depends upon the VC for its development and survival and the critical importance of this association is apparent from targeted ablation of the VC, which leads to loss of GC viability (Twell 1995). The GC and VC both undergo structural modifications, but the nature of morphogenetic events and their timing vary during pollen development. For example, the VC nucleus becomes highly lobed in mature pollen of bicellular and tricellular species (Russell and Cass 1981; McConchie et al. 1985; Mogensen 1986; Niu et al. 1999). We observed irregularities in the VC nuclear envelope in bicellular stage pollen that progressed to highly irregular lobed forms in tricellular stages (Fig. 2; Movie 10–11). This modulation of VC nucleus shape has recently been attributed to KAKU4, a putative component of the nuclear lamina (Goto et al. 2020). KAKU4-mediated deformation of the VC nucleus is proposed to facilitate its entry into the pollen tube as migration order of the VC nucleus and SCs is often reversed in *kaku4* mutants (Goto et al. 2020).

In early bicellular pollen, we observed re-shaping of Arabidopsis GCs to spheroidal after detachment from the pollen wall (Figs. 1c, i; S1, S2; Movie 1), as reported for other angiosperms (Sanger and Jackson 1971; Brighigna et al. 1981; Theunis et al. 1985; Tanaka 1988; Yu and Russell 1992; Eva et al. 2006). In nearly all species examined GC morphology is initially spheroidal when internalised within the VC and elongated/fusiform in later stages, but transitional states are not generally documented (Table S1 and S2). At mid-bicellular pollen stage, we observed partial elongation of the GC (Figs. 1d, 4b–c), like GCs of some other species (Zhou and Yang 1991; Niu et al. 1999; Zee and Ye 2000; Kliwer and Dresselhaus 2010; Oh et al. 2010). In summary, the overall pattern of GC body morphogenesis in Arabidopsis follows that of other angiosperms (Fig. S12, Table S1 and S2).

### The adaptive role of GC elongation

The elongated shape of the GC was described over a century ago (Ducker and Knox 1985) and is recognised as a conserved character of angiosperms that shed either bicellular or tricellular pollen (Table S1 and S2). The biological role of germ cell lineage elongation is presumed to be an adaptation to ease passage through the confines of the pollen tube, while the distinctive cortical cage of axial MTs is thought to play an important role in shaping and maintaining GC cell morphology (reviewed in Palevitz and Tiezzi 1992; Zhang et al. 1995; Russell and Strout 2005). For example, treatment of *Haemanthus katharinae* (Sanger and Jackson 1971) or *Galanthus nivalis* pollen (Heslop-Harrison et al. 1988) with anti-MT drugs reverted the shape of GCs to ovoid or spheroidal. Moreover, isolated GCs or SCs become spheroidal after release from pollen or pollen tubes and their distinctive MT organisation is lost (Tanaka 1988; Theunis et al. 1992; Hirano and Hoshino 2010; Kliwer and Dresselhaus 2010). However, isolation of SCs into media with low osmotic potential in *Gerbera* maintained the spindle shape, indicating that osmotic conditions as environmental factors are also important for maintenance of GC and SC shape (Southworth and Knox 1989). The partial reversion of elongated germ cell shape in vivo further suggests that structural features in addition to the distinctive MT array also play a role in maintaining cell shape (Sanger and Jackson 1971; Hirano and Hoshino 2010).

During entry of the GC or SCs into the confines of the pollen tube, there can be extensive changes in shape involving further elongation of both the germline cells and the VC nucleus (Åström et al. 1995; Ge et al. 2011; Lalanne and Twell 2002). Prior elongation of the GC and SCs may be energetically favourable to support rapid pollen germination and transport of the male germ unit. In exceptional cases, the GC is spheroidal in mature pollen but undergoes dramatic re-shaping and elongation before entering the pollen tube (Pandolfi et al. 1993; Zhang et al. 1995). In *Gagea lutea* (Liliaceae), re-shaping of the GC is accompanied by reorganisation of the cortical MT arrays into thick bundles arranged longitudinally (Zhang et al. 1995). Such cases have been suggested to be an adaptation to the energy demands of maintaining a spindle-shaped GC with a well-organised cytoskeleton (Pacini and Hesse 2002).

A further potential role for elongation of the GC prior to mitotic division is that it provides adequate space for spindle assembly and chromosome segregation in a cell of restricted volume. Spindle assembly and chromosome congression present challenges in the confines of the pollen tube. These events are compounded in species with large chromosomes and/or polyploid genomes, wherein irregular chromosome arrangements and plasticity in the geometry of GC division have been described (Palevitz and Cresti 1989; Sax and O’Mara 1941).

### DUO1 has a specific role in GC morphogenesis

**Fig. 8:**
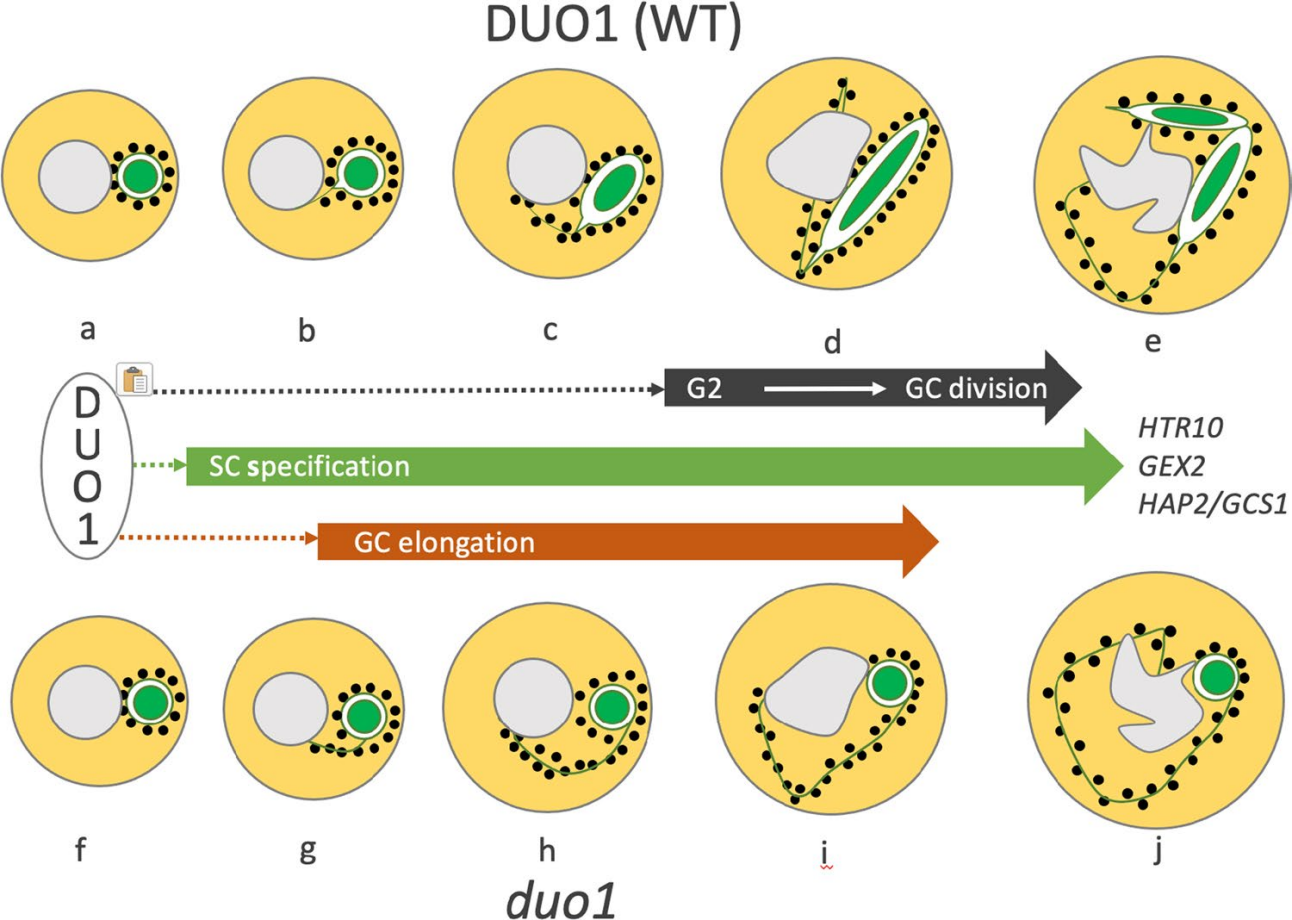
Summary of the roles of DUO1 in *Arabidopsis* germline development. The GC forms a cytoplasmic extension and elongates during bicellular pollen development (**a**–**d**). DUO1 regulates both GC division (black arrow) and SC specification (green arrow). In mutant *duo1* pollen, the GC fails to elongate and divide but forms a cytoplasmic extension (**f**–**j**), like the GC in wild-type pollen (**a**–**e**). DUO1, therefore, has a key role in GC elongation (orange arrow). Examples of direct DUO1-target genes are shown; *HTR10, HISTONE THREE RELATED 10; GEX2, GAMETE EXPRESSED 2; HAP2/GCS1, HAPLESS 2/GENERATIVE CELL SPECIFIC 1*

The requirement of DUO1 for complete GC morphogenesis adds to its established role as a key transcription factor regulating GC division and gamete differentiation (Fig. 8). The analysis of GCs deficient in DUO1 or CDKA;1-function highlights a coordinating role for DUO1 in these processes (Figs. 1, 3, 5). In contrast to *duo1* alleles, CDKA;1-function mutants do not affect GC elongation even though GC division does not occur in pollen (Iwakawa et al. 2006; Nowack et al. 2006; Kim et al. 2008; Gusti et al. 2009), (Figs. 7; S10–12). The uncoupling of GC elongation and cell cycle progression in *cdka;1* and *fbl17* mutants indicates that while these processes are not co-dependent, they are coordinated by DUO1 as a germline-specific regulator. Moreover, the complete lack of GC body elongation in multiple alleles of *duo1* (Figs. 4, 5) highlights the essential role for DUO1 in this process and the importance of DUO1-independent pathways in the formation and growth of the GC cytoplasmic projection.

Given that elongation of the GC body is closely linked to microtubule organisation, DUO1-dependent candidate genes with a structural or regulatory roles in MT assembly or dynamics may be involved. Although tubulin genes are not among known DUO1-target genes identified in Arabidopsis, a potential regulator is the mitogen activated protein kinase kinase kinase, MAPKKK20 (Borg et al. 2011). MAPKKK20 has been reported to be involved in cortical microtubule functions in roots and could therefore be linked to MT dynamics involved in the initiation and or maintenance of GC elongation (Benhamman et al. 2017). Another candidate DUO1-target protein is the tonoplast localised male germline-specific aquaporin TIP5;1, which has been suggested to facilitate equilibration of cellular water during pollen dehydration/rehydration or during vacuolar rearrangements in germ cells (Wudick et al. 2014). Given that germ cells possess limited resistance to osmotic stresses because of their rudimentary cell walls, osmotic adjustment may also be required to maintain germ cell shape in addition to the mechanical support provided by the cortical MT system.

### DUO1-independent formation of the GC cytoplasmic projection

There is limited understanding of how GC projections develop, although MTs extend from the GC body into the cytoplasmic projection and may be involved in their initiation or growth (Palevitz and Tiezzi 1992; Russell et al. 1996; Oh et al. 2010; McCue et al. 2011). As neither of these processes were clearly affected in DUO1 or in CDKA;1-function mutants (Figs. 5, 6, 7), they appear to operate independently of DUO1 control and cell cycle progression. We observed that a single cytoplasmic projection forms while the GC is spheroidal (Figs. 1c, i; S3), in agreement with detailed observations in *Plumbago* (Russell et al. 1996). Most Arabidopsis GCs (95.1%) formed a single cytoplasmic projection, while the remainder formed a second shorter projection at the opposite end of the elongating GC (Table S7; Fig. 3). It is notable that over 90% of undivided GCs in *duo1* mutants formed a single projection (Table S7), suggesting that growth of the second projection depends on axial elongation of the GC. Continued growth of the cytoplasmic projection and GC elongation occur concomitantly in wild-type pollen (Fig. 1i–k) but these events are uncoupled in *duo1* pollen, suggesting that they are independently regulated. We also observed alignment of polar elongation of the GC with the origin of the cytoplasmic projection (Figs. 3a–c, 5b–e and S5a–d). This feature, also observed in ultrastructural (Russell et al. 1996) and live cell (Oh et al. 2010) studies, suggests that initiation of the GC projection is linked to an emergent cortical MT system that initiates GC re-shaping and subsequently provides mechanical stability to the developing germ cell lineage.

## Conclusions

Our results show that formation of the GC cytoplasmic projection and axial elongation of the GC body are independently regulated processes. The uncoupling of these morphogenetic features in allelic *duo1* mutants establishes their independent regulation and the requirement of DUO1 for elongation of the GC. Our data also imply that association of the GC with the VC nucleus, as a precursor of the male germ unit, is largely dependent on the cytoplasmic projection and does not require elongation of the GC body. We further show that formation and growth of the cytoplasmic projection is not dependent upon DUO1 or CDKA;1-dependent cycle progression and although closely coupled, GC elongation and division are independently regulated.

The discovery of a previously unknown role for DUO1 in angiosperm pollen also reflects the ancestral role of DUO1 in sperm differentiation in the land plant lineage (Higo et al. 2018). Loss of DUO1 in the bryophyte *Marchantia polymorpha* blocks spermatid differentiation, resulting in failure of nuclear elongation and the absence of a flagellar apparatus. Moreover, two antheridia-specific ⍺-tubulin isoforms connect the DUO1 gene regulatory network to the MT cytoskeleton in *Marchantia* (Higo et al. 2018). Additionally, it has been suggested that the distinctive GC MT system of angiosperms may be derived from elements of the motility apparatus present in ancestors with flagellated sperm (Palevitz and Tiezzi 1992; Southworth and Cresti 1997). As tubulin genes were not among Arabidopsis DUO1-targets (Borg et al. 2011) and flagella have been lost, a possible scenario is that rewiring of the DUO1 network may have occurred to support modified cell morphogenesis in angiosperms.

## Supplementary Information

Below is the link to the electronic supplementary material.

Supplementary file1 (DOCX 9426 kb)

Supplementary file2 (DOCX 12 kb)

Supplementary file3 (AVI 2452 kb)

Supplementary file4 (AVI 1583 kb)

Supplementary file5 (AVI 2023 kb)

Supplementary file6 (AVI 1560 kb)

Supplementary file7 (AVI 2276 kb)

Supplementary file8 (AVI 1739 kb)

Supplementary file9 (AVI 1793 kb)

Supplementary file10 (AVI 1679 kb)

Supplementary file11 (AVI 1889 kb)

Supplementary file12 (AVI 1509 kb)

Supplementary file13 (AVI 1448 kb)

Supplementary file14 (AVI 849 kb)
