# Supplementary material for: Genetic control of generative cell shape by DUO1 in Arabidopsis
Source: Plant Reprod. 2023 Apr 6;36(3):243–54. doi: 10.1007/s00497-023-00462-x (PMC10363056; doi:10.1007/s00497-023-00462-x)
Supplement: Supplementary file 1 — Supplementary file1 (DOCX 9426 kb) [file 497_2023_462_MOESM1_ESM.docx]

**Supplementary Information (SI)**

**Article:** **Genetic control of generative cell shape by DUO1 in *Arabidopsis***

Authors: Abdur Rauf ^1,2^, Hoda Khatab^1,3^, Michael Borg^1,4^ and David Twell^1*^

^1^Department of Genetics and Genome Biology, University of Leicester, Leicester, LE1 7RH, UK.

^2^Department of Botany, Garden Campus, Abdul Wali Khan University Mardan, Mardan, Pakistan.

^3^Department of Botany, Faculty of Science, University of Omer Al-Mukhtar, Al-Baida, Libya.

^4^Department of Algal Development and Evolution, Max Planck Institute for Biology Tübingen, Max-Planck-Ring 5, 72076, Tübingen, Germany.

*Corresponding author e-mail – [twe@le.ac.uk](mailto:twe@le.ac.uk)

**Table S1.** Cytological evidence for generative cell morphogenesis in tricellular pollen species. The information summarised is collected using various techniques. The GC was shown to be spindle-shaped (fusiform, F) or tear drop-shaped (lachrymiform, L). IF, Immunofluorescence; α, Alpha; FM, fluorescence microscopy; LM, Light microscopy; FCM, flow cytometry; TEM, transmission electron microscopy; CLSM, confocal laser scanning microscopy; DAPI, 4′,6-diamidino-2-phenylindole; MT, microtubules; YFP, yellow fluorescent protein; TBO, toluidine blue; DiOC6, 3,3-dihexyloxacarbocyanine iodide.

| **Species** | **Stages analysed** | **GC Shape** | **Technique(s)** | **Reference** |
| --- | --- | --- | --- | --- |
| *Hordeum vulgare* | BCP to MPG | F | TEM | Charzynska *et al.,* 1988 |
| *Oryza sativa* | 5-Stages MS to MPG | F | Anti-α-tubulin IF, CLSM | Ze and Ye, 2000 |
| *Plumbago zeylanica* | MS to MPG | L | TEM- serial sectioning | Russell and Strout, 2005 |
| *Brassica napus* | MS to MPG | F | TEM, FM-DAPI, LM | Murgia *et al.,* 1991 |
| *Zea mays* | GC to SC in pollen tube | L | FM-DAPI, CLSM, α-tubulin-YFP | Kliwer and Dresselhaus, 2010 |
| *Brassica napus* | MS to SCs | F | Anti-α-tubulin IF, CLSM, DIC, FM-DAPI, FM-DiOC_6_ | Dubas *et al.,* 2012 |
| *Euphorbia dulcis* | MS to MPG | F | TEM | Murgia *et al.,* 1986 |
| *Euphorbia dulcis* | MS to SCs | L | TEM | Murgia and Wilms, 1987 |
| *Linaria vulgaris* | MS to SCs | L | SEM and TEM | Cresti *et al.,* 1988 |
| *Abelia spathulata Campsis grandiflora Erythrina variegata Limonium sinuatum* *Tecoma capensis* | MS to SCs | L | FM-DAPI, TBO and FM-DiOC_6_ | Saito *et al.,* 2002 |
| *Lolium perenne* | Isolated GC and SCs | F | FCM, TEM, CLSM | Van der Maas *et al.,* 1994 |

**Table S2.** Cytological evidence for generative cell morphogenesis in bicellular pollen species. The information summarised is collected using various techniques. The GC was shown to be spindle-shaped (fusiform, F) or tear drop-shaped (lachrymiform, L). IF, Immunofluorescence; α, Alpha; β, beta; γ, gamma; TEM, transmission electron microscopy; SEM, scanning electron microscopy, CLSM, confocal laser scanning microscopy; DAPI, 4′,6-diamidino-2-phenylindole; YFP; yellow fluorescent protein; TBO, toluidine blue; DiOC6, 3,3-dihexyloxacarbocyanine iodide; FISH, fluorescence in situ hybridisation; FCM, flow cytometry; MT, Microtubules; IIF, indirect immunofluorescence; MFs, microfilaments; RF-FS, rapid freeze fixation and freeze substitution; 5Mc, 5-methylcytosine; ACH4, acetylated histone 4; HDT1 histone deacetylase 1; LM, Light microscopy.

| Plant name | Family | Developmental stages analysed | GC shape | Technique(s) | Reference |
| --- | --- | --- | --- | --- | --- |
| *Nicotiana tabacum* | Solanaceae | GC to SCs in pollen tube | F | Anti-α-tubulin-IF | Palevitz, 1993 |
| *N. tabacum* | Solanaceae | GC to SCs in pollen tube | F | Anti-α-tubulin-IF, FM-aniline blue and FM-DAPI-CLSM | Laitiainen *et al.,* 2002 |
| *N. tabacum*  *N. alata* | Solanaceae | GC development and division inside pollen tube | F | Anti-α-tubulin-IF, Rhodamine-phalloidin MFs stain, FM-DAPI | Äström *et al.,* 1995 |
| *N. tabacum* | Solanaceae | GC to SCs in pollen tube | L | Serial TEM | Yu and Russell, 1994 |
| *N. tabacum* | Solanaceae | MS to fertilization | L | LM, CLSM | Oh *et al.,* 2010 |
| *N. tabacum* | Solanaceae | GC division in pollen tube | L | Anti-γ and β-tubulins IF | Palevitz *et al.,* 1994 |
| *N. alata* | Solanaceae | GC division in pollen tube | L | RF-FS-TEM | Lancelle *et al.,* 1987 |
| *N. alata*  *Impatiens wallerana* | Solanaceae  Balsaminaceae | In vitro germination of MPG and GC in pollen tube | L | RF-FS-TEM | Cresti *et al.,* 1987 |
| *N. tabacum* | Solanaceae | GC division in pollen tube | F | IF, FM-DAPI | Cai *et al.,* 2005 |
| *Hippeastrum vitatum* | Liliaceae | Mature pollen | F | 3-D reconstruction and TEM | Mogenson, 1986 |
| *Hyacinthus orientalis* | Asparagaceae | GC to SC in pollen tube | F | Anti-α-tubulin-IF, Rhodamine-phalloidin F-actin stain, TEM, CLSM | Del Casino *et al.,* 1992 |
| *H. orientalis* | Asparagaceae | MPG, GC division in pollen tube | F | 5-Bromouracil IF, FISH, FM-DAPI, CLSM | Zienkiewicz *et al.,* 2011 |
| *Medicago sativa* | Fabaceae | GC in pollen tube | L | 3-D reconstruction, TEM | Zhu *et al.,* 1990 |
| *Medicago sativa* | Fabaceae | GC to SCs in pollen tube | F | 3-D reconstruction, TEM | Shi *et al.,* 1991 |
| *Rhododendron anagalliflorum, R.laetum, R.lochae* | Ericaceae | Early to late BCP | L | 3-D reconstruction, TEM, LM | Theunis *et al.,* 1985 |
| *Allium sp.*  *Crinum, Gladiolus, Camellia,*  *Thea, Zephyranthes* | Amaryllidaceae  Theaceae | GC in pollen tube | L | In vivo and phase contrast | Ota, 1957 |
| *Lilium longiflorum* | Liliaceae | MS to late BCP | F | LM, FM of acetocarmine treated GC | Tanaka, 1988 |
| *Tillandsia seleriana* | Bromeliaceae | MS to late BCP | L | TEM | Brighigna *et al.,* 1981 |
| *Gagea lutea* | Liliaceae | GC to SCs in pollen tube | L | Video microscopy, DIC, CLSM, 3-D reconstruction | Zhang *et al.,* 1995 |
| *Leucojum aestivum* | Amaryllidaceae | Microsporogenesis, microgametogenesis and in vitro pollen tube | L | LM, FM | Ekici and Dane, 2012 |
| *Tropaeolum majus* | Tropaeolaceae | MS to SCs in pollen tube | L | FM-DAPI, TEM | Niu *et al.,* 1999 |
| *Haemanthus katherinae* | Amaryllidaceae | MS to anthesis | L | LM, TEM | Sanger and Jackson, 1971b |
| *Tillandsia seleriana* | Bromeliaceae | Early to late BCP | L | LM, TEM | Eva *et al.,* 2006 |
| *Cyrtandra pendula* | Gesneriaceae | Early to late BCP | F | TEM | Luegmayr, 1993 |
| *Populus deltoides* | Salicaceae | GC in MPG to SCs in pollen tube | L | FM, TEM | Rougier *et al.,* 1991 |
| *T. virginiana,*  *N. tabacum Rhododendron laetum* | CommelinaceaeSolanaceae Ericaceae | GC in pollen tube | L | Anti-actin-IF, hoechst 33258 and N-phenylenediamine nuclear staining | Palevitz and Liu, 1992 |
| *Cymbidium goeringii* | Orchidaceae | Early to anthesis | L | TEM, 3-D serial reconstruction and quantitative cytology | Yu and Russell, 1992 |
| *T. virginiana* | Commelinaceae | GC and SC in pollen tube | L | Anti-α-tubulin-IF, Rhodamine-phalloidin F-actin stain and FM-DAPI | Palevitz and Cresti, 1988 |
| *T. virginiana* | Commelinaceae | GC in pollen tube | L | Anti-α-tubulin-IF, Rhodamine-phalloidin F-actin stain and FM-DAPI | Palevitz and Cresti, 1989 |
| *Ornithogalum virens* | Asparagaceae | GC in MPG to SCs in pollen tube | L | Anti-α-tubulin IF, CLSM | Banas *et al.,* 1996 |
| *Tradescantia reflexa* | Commelinaceae | GC in MPG | L | TEM | Noguchi and Ueda, 1990 |
| *Helleborus foetidus* | Ranunculaceae | GC in MPG (hydrated and dehydrated) to pollen tube | L | FM-DAPI, optical sectioning microscopy | Heslop-Harrison *et al.,* 1986 |
| *Petunia hybrida* | Solanaceae | GC in MPG to pollen tube | L | Phase contrast and 3-D reconstruction | Wagner and Mogensen, 1988 |
| *Tillandsia caput-medusae* | Bromeliaceae | GC in early to late BCP | L | TEM | Brighigna *et al.,* 1981 |
| *Allemanda neriifolia* | Apocynaceae | GC in pollen tube | F | Anti-α-tubulin IF | Zee, 1992 |
| *Eucalyptus globulus* | Myrtaceae | MPG | F | LM, SEM, TEM | Eliseu and Dini, 2008 |
| *Luehea divaricata* | Malvaceae | Microsporogenesis and microgametogenesis | F | TEM | Lattar *et al.,* 2012 |
| *Chlorophytum comosum* | Asparagaceae | MS to MPG | F | TEM | Schröder, 1986 |
| *Gasteria verrueosa* | Asphodelaceae | MS to MPG | F | TEM | Schröder, 1985 |
| *Convallaria majalis* | Asparagaceae | GC division in pollen tube | L | Anti-α-tubulin IF, DAPI, CLSM | Del Casino *et al.,* 1999 |
| *N. tabacum* | Solanaceae | GC division in pollen tube | F | Video microscopy, Anti-α-tubulin IF, TEM, CLSM | Theunis *et al.,* 1992 |
| *Gasteria verrucosa* | Asphodelaceae | MS to SCs in pollen tube | F | Microspectrophotometry, Anti-α-tubulin-IF, TEM | Van Lammeren *et al.,* 1985 |
| *Cyrtanthus mackenii* | Amaryllidaceae | GC division in pollen tube | F | FCM, FM-DAPI, Anti-α-tubulin IF | Hirano and Hoshino, 2010 |
| *Cyphomandra betacea* | Solanaceae | GC in pollen tube | L | Light (DAPI) and TEM | Hu and Yu, 1988 |
| *Hippeastrum vittatum* | Liliaceae | GC early to late buds | F | Anti-α-tubulin IF and 3-D reconstruction | Zhou and Yang, 1991 |
| *Allemanda neriifolia* | Apocynaceae | Isolated GC from pollen tube | F | Anti-α-tubulin IF of germline MTs | Zee, 1991 |
| *Gossypium hirsutum* | Malvaceae | Mature germinating pollen | L | LM, TEM | Jensen *et al.,* 1968 |
| *Papaver rhoeas* | Papaveraceae | MPG | F | TEM | Cresti *et al.,* 1990 |
| *Hyacinthus orientalis* | Asparagaceae | MPG and GC division in pollen tube | L | Immunolocalisation of 5Mc, ACH4, HDT1 and FM-DAPI | Kozłowska *et al.,* 2016 |
| *Vitis vinifera* | Vitaceae | MPG | L | SEM and TEM | Cresti and Ciampolini, 1999 |
| *Fritillaria stribrnyi* | Liliaceae | Microsporogenesis to GC division in pollen tube | L | LM-acetic-orcein, lactophenol-aniline blue | Kartal, 2015 |
| *Endymion non-scriptus* | Asparagaceae | MPG | L | TEM | Burgess, 1970 |
| *Trillium kamtschaticum* | Trilliaceae | Early MS to MPG | F | TEM | Takahashi, 1987 |
| *Tulipa gesneriana* | Liliaceae | Early MS to MPG | L | LM | Tanaka and Ito, 1981 |
| *Populus deltoides* | Salicaceae | GC in MPG to SC in pollen tube | L | FM-DAPI, LM, TEM | Rougier *et al.,* 1991 |
| *Acacia retinodes* | Fabaceae | Early to GC in MPG | F | LM | McCoy and Knox, 1988 |
| *Crocus biflorus* | Iridaceae | MPG | F | FM-DAPI, LM, TEM | Caiola *et al.,* 1993 |
| *Hymenocallis littoralis* | Amaryllidaceae | Mature pollen | F | SYBR Green I FM and TEM | Asai *et al.,* 2013 |

**Table S3.** Transgenic pollen markers used in this study. The markers are male germline-specific (1 – 6), vegetative cell-specific (7) or decorate both vegetative and male germline cells (8).

| Marker | | Marker expression in germline | | | Marker expression in vegetative cell | | Reference |
| --- | --- | --- | --- | --- | --- | --- | --- |
|  |  | Plasma membrane | Cytoplasm/Microtubules | Nucleus | Nucleus | Nuclear membrane |  |
| 1 | TET11-GFP | √ | **×** | **×** | **×** | **×** | Boavida *et al.,* 2013 |
| 2 | DUO1:TET11-tdTomato | √ | **×** | **×** | **×** | **×** | This study |
| 3 | MBD10:TET11-tdTomato | √ | **×** | **×** | **×** | **×** | This study |
| 4 | HTR10:GFP-TUA6 | **×** | √ | **×** | **×** | **×** | Ueda *et al.,* 1999;  Khatab, 2012 |
| 5 | HTR10:H2B-RFP | **×** | **×** | √ | **×** | **×** | This study |
| 6 | DUO1:H2B-tdTomato | **×** | **×** | √ | **×** | **×** | Borg *et al.,* 2009 |
| 7 | LAT52:RanGAP-tdTomato | **×** | **×** | **×** | **×** | √ | Rose & Meier, 2001; this study |
| 8 | DUO3:H2B-tdTomato | **×** | **×** | √ | √ | **×** | Brownfield *et al.*, 2009 |

**Table S4.** Transgenic markers and their expression in wild type and mutant pollen. The germline markers TET11-GFP, HTR10:GFP-TUA6 and DUO1:TET11-tdTomato are expressed in wild type and in *fbl17* and *cdka;1* pollen. MBD10:TET11-tdTomato, DUO1:H2B-tdTomato, LAT52:RanGAP-tdTomato and DUO3:H2B-tdTomato are all expressed in mutant pollen of all *duo1* alleles. ^1^ HTR10:H2B-RFP was used to differentiate wild type GC/SCs from undivided mutant GCs in *duo1*. ^2^MBD10:TET11-tdTomato has comparatively higher expression in *duo1* mutant GCs than in WT. The method used to introduce markers into wild type and mutant lines is indicated.

| Genotype | Germline markers | VC markers | Method  (SC, sexual cross; T, transformed) |
| --- | --- | --- | --- |
| WT | TET11-GFP, HTR10:GFP-TUA6, DUO1:H2B-tdTomato |  | SC |
|  | DUO1:TET11-tdTomato  HTR10:H2B-RFP  MBD10:TET11-tdTomato^2^  DUO3:H2B-tdTomato  LAT52:RanGAP-tdTomato | DUO3:H2B-tdTomato,  LAT52:RanGAP-tdTomato | T |
| *duo1-1^+/-^*  *duo1-2^+/-^*  *duo1-3^+/-^*  *duo1-4^+/-^* | TET11-GFP, DUO1:H2B-tdTomato |  | SC |
|  | DUO1:TET11-tdTomato, HTR10:H2B-RFP^1^  MBD10:TET11-tdTomato^2^  DUO3:H2B-tdTomato,  LAT52:RanGAP-tdTomato | DUO3:H2B-tdTomato  LAT52:RanGAP-tdTomato | T |
| *fbl17^+/-^* | TET11-GFP, HTR10: GFP-TUA6 |  | SC |
|  | DUO1:TET11-tdTomato |  | T |
| *cdka;1^+/-^* | TET11-GFP, HTR10: GFP-TUA6 |  | SC |
|  | DUO1:TET11-tdTomato |  | T |

**Table S5.** Mutant alleles used in this study. The four *duo1* mutant alleles are illustrated in the schematic diagram. Nucleotide positions of mutations or insertions are indicated in the red triangles for each allele. Introns (lines) join exons (boxes) and the R2R3 MYB domain is filled (black). Start and stop codons are indicated.

| Mutant allele | Accession | Locus | Mutagen and lesion | Reference |
| --- | --- | --- | --- | --- |
| *cdka;1* | Col-0 | AT3G48750 | T-DNA insertion +1816 bp (SALK_106809) | Iwakawa et al., 2006; Nowack et al., 2006 |
| *duo1-1* | No-0 | At3G60460 | EMS, C>T +812 bp | Durbarry et al., 2005;  Rotman et al., 2005 |
| *duo1-2* | C24 | AT3G60460 | Gamma-rays, 14 bp insertion (TTGGGTGATGAAGA) +672 bp | Rotman et al., 2005 |
| *duo1-3* | Ler-0 | AT3G60460 | Gene Trap Ds insertion +198 bp (GT_5_18345) | NASC ID: N175915 |
| *duo1-4* | Col-0 | AT3G60460 | EMS, C>T +545 bp | Borg et al., 2014 |
| *fbl17* | Col-0 | AT3G54650 | T-DNA insertion +1278 bp (GABI_170E02) | Kim et al., 2008 |
| 198  *duo1-2* (TTGGGTGATGAAGA)  *duo1-1* (C>T)  *duo1-4* (C>T)  *duo1-3* (GT *Ds*)  1  188  286  547  620  672  852  Start  Stop  1063  545 | | | | |

**Table S6.** Developmental analysis of GC morphogenesis. Dimensions of the GC body in TET11-GFP/HTR10:H2B-RFP labelled pollen were analysed with Image J at three developmental stages. WT GCs were scored based on positive HTR10 marker expression and/or elongation of half of the pollen population at mid bicellular (-6) and late bicellular (-4 to -5) bud stages, while HTR10-negative pollen (*duo1*) contained a GC with a round profile. Mean (*M*) and standard deviation (*SD*) were calculated from *N* individual WT and *duo1-4* plants in a minimum of three separate experiments, where 4-40 pollen were scored per plant per stage. Aspect ratio = [Major axis]/[Minor axis] for the fitted ellipse; Circularity = 4π × [Area]/[Perimeter]^2^, with a value of 1.0 indicating a perfect circle and values approaching 0.0 indicating an increasingly elongated shape (<https://imagej.nih.gov/ij/index.html>).

|  | | Developmental stages | | | | | |
| --- | --- | --- | --- | --- | --- | --- | --- |
|  |  | Early bicellular | | Mid bicellular | | Late bicellular | |
|  |  | WT | *duo1* | WT | *duo1* | WT | *duo1* |
| Major axis (µm) | *M* | 4.97 | 4.95 | 7.05 | 4.89 | 10.29 | 4.50 |
|  | *SD* | 0.29 | 0.26 | 0.70 | 0.25 | 1.01 | 0.14 |
| Minor axis (µm) | *M* | 4.87 | 4.91 | 4.09 | 4.82 | 3.35 | 4.44 |
|  | *SD* | 0.27 | 0.29 | 0.31 | 0.27 | 0.25 | 0.13 |
| Aspect ratio | *M* | 1.02 | 1.01 | 1.72 | 1.01 | 3.07 | 1.01 |
|  | *SD* | 0.00 | 0.01 | 0.17 | 0.01 | 0.20 | 0.00 |
| Circularity | *M* | 1.00 | 1.00 | 0.73 | 1.00 | 0.45 | 1.00 |
|  | *SD* | 0.00 | 0.00 | 0.06 | 0.00 | 0.04 | 0.00 |
|  | *N* | 3 | 3 | 4 | 5 | 6 | 3 |

**Table S7**. Frequency of germline cytoplasmic projections in wild type (WT) and *duo1* mutant pollen in GC and SC stage flower buds. Mean, standard deviation (SD) and percentage data are shown for plants (*N* = 3) of each genotype, where 58 – 300 pollen were scored per plant for WT (HTR10:GFP-TUA6) or *duo1* (TET11-GFP). In WT pollen at SC stage, one SC always possessed a long cytoplasmic projection, while the second SC lacked a projection (87 %) or had a shorter projection (13 %). In chi-squared tests the proportion of pollen with one or two cytoplasmic projections was not significantly different between WT and *duo1* alleles at GC stage (P=0.51) or at SC stage (P=0.29). An increase in the proportion of pollen with two cytoplasmic projections between GC and SC stage was observed for all genotypes, but this was only significant (P<.05) for *duo1-1* (P=.012).

| Genotype | Number of cytoplasmic projections | Developmental stage | | | |
| --- | --- | --- | --- | --- | --- |
|  |  | GC | | SC | |
|  |  | Mean (SD) | % | Mean (SD) | % |
| WT (*N* = 3) | 1 | 91.3 (26.3) | 95.1 | 95.7 (48.0) | 87.0 |
|  | 2 | 4.7 (0.6) | 4.9 | 14.0 (9.7) | 13.0 |
| *duo1-1* (*N* = 3) | 1 | 158.7 (119.4) | 97.3 | 143.0 (109.4) | 91.1 |
|  | 2 | 4.3 (0.6) | 2.7 | 14.0 (14.8) | 8.9 |
| *duo1-3* (*N* = 3) | 1 | 107.0 (11.8) | 96.7 | 178.3 (80.6) | 92.9 |
|  | 2 | 3.7 (0.6) | 3.3 | 13.7 (15.0) | 7.1 |

**
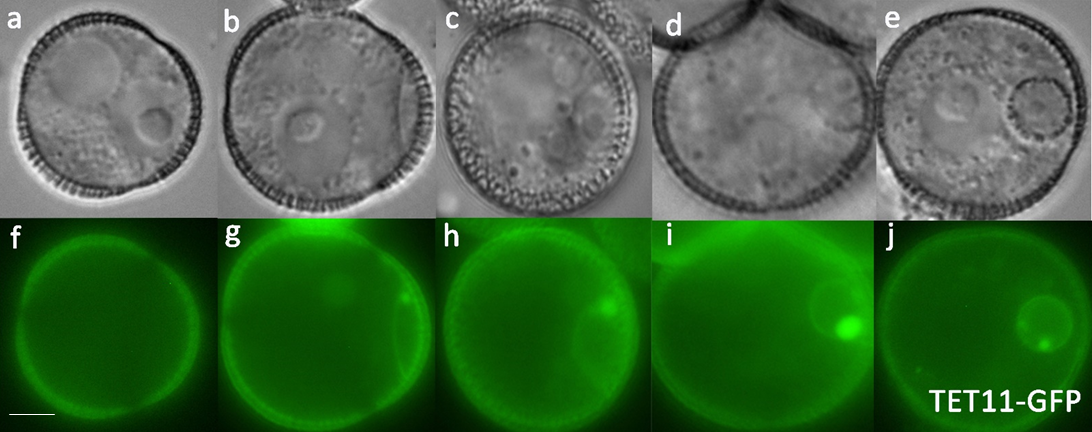
**

**Fig. S1.** Formation and detachment of the GC from the pollen wall. Corresponding DIC (a-e) and fluorescence (f-j) images of wild type pollen marked with TET11-GFP decorating the plasma membranes between the VC and GC. (a) polarised microspore, (b-c) early GC attached with curved wall, (d) later GC with domed wall, (e) recently detached round GC. Scale bar = 5 µm.

**
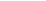
**


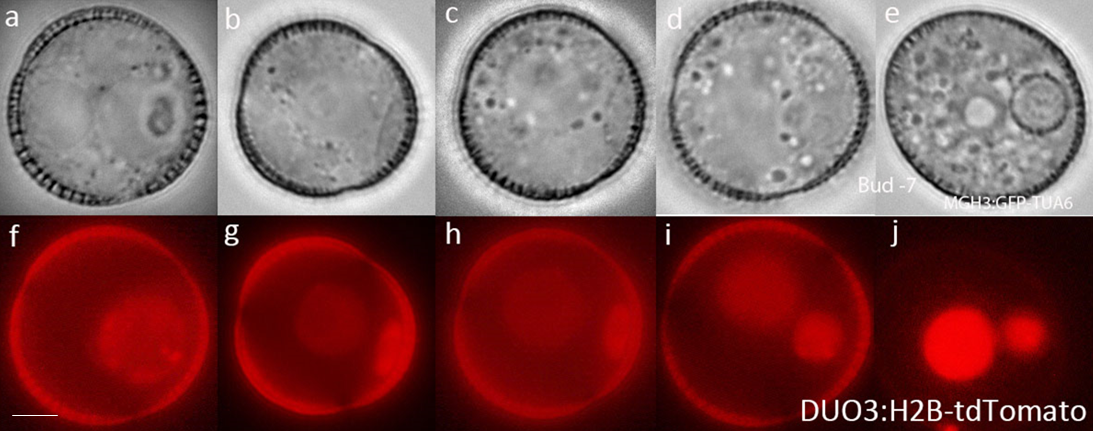


**Fig. S2.** Microspore to early bicellular pollen stages marked with DUO3:H2B-tdTomato. Corresponding DIC (a-e) and fluorescence (f-j) images of developing pollen marked with DUO3:H2B-tdTomato. (a,f) polarized microspore with a large RFP-labelled nucleus (f). (b) newly formed lenticular GC attached to the pollen wall with labelled compact GC nucleus and larger diffuse VC nucleus (g). The GC profile changes from lenticular (b-c) to round while attached to the pollen wall (d) and then remains round early after detachment (e). Scale bar = 5 µm.

**
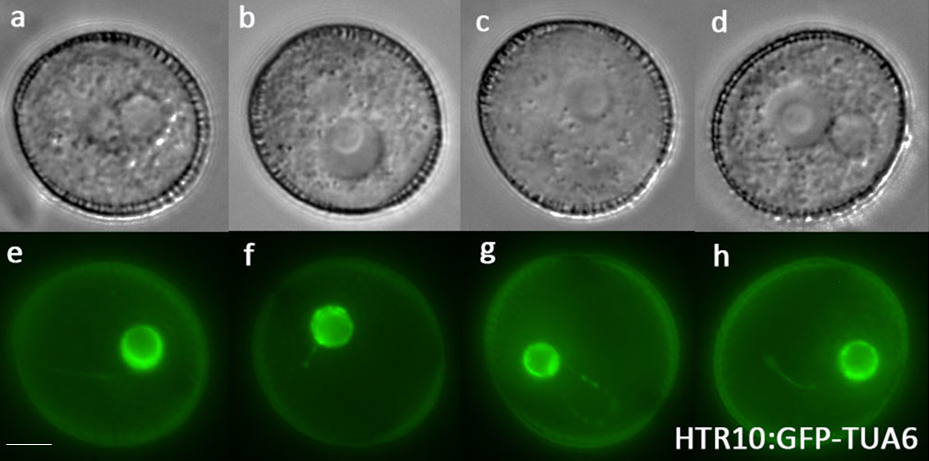
**

**Fig. S3.** The GC cytoplasmic projection forms prior to elongation of the GC body. Corresponding DIC (a-d) and fluorescence (e-h) images of wild type pollen at early bicellular stage labelled with HTR10:GFP-TUA6. At this stage the GC appears round in profile and closely associated with the vegetative cell nuclear membrane but possesses a fine cytoplasmic projection. Scale bar = 5 µm.

**
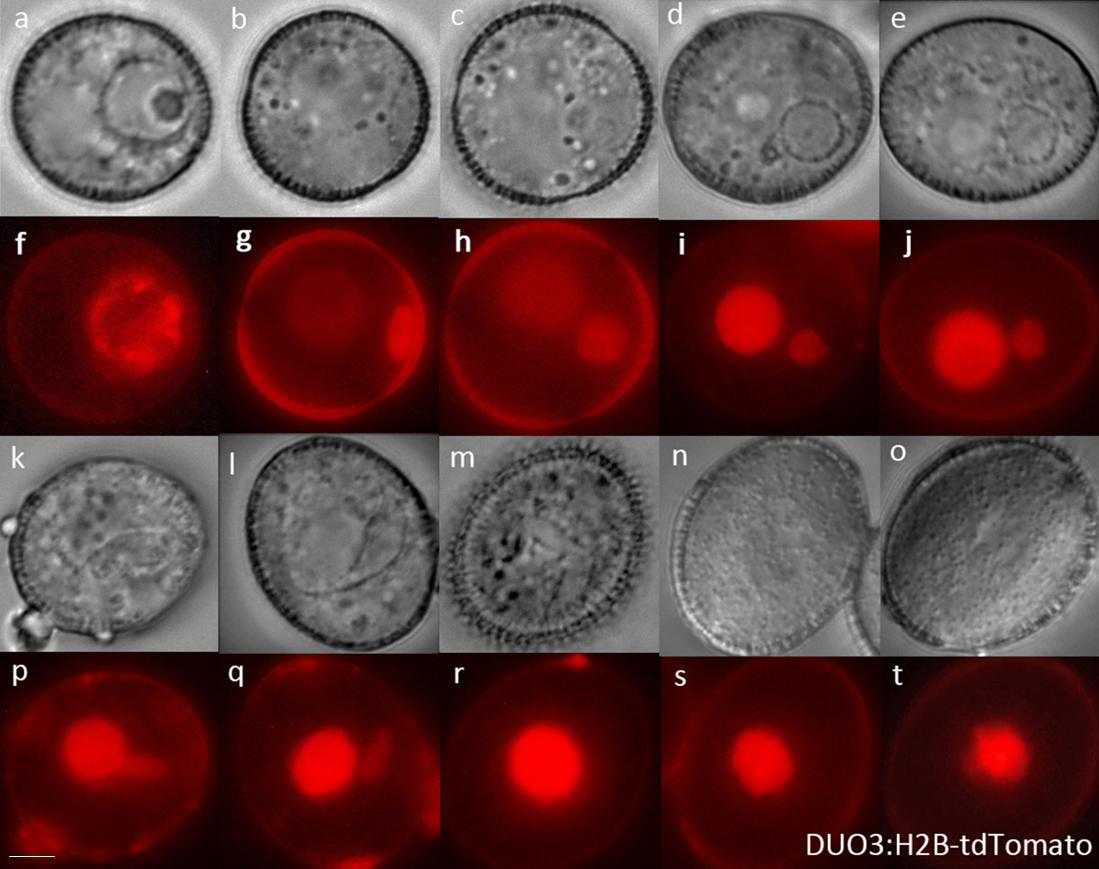
**

**Fig. S4.** Morphogenesis of the vegetative cell nucleus in wild type pollen. Corresponding DIC (a-e) and fluorescence images of developing pollen with DUO3:H2B-tdTomato labelled nuclei. (a, f) polarized microspore with large RFP-labelled nucleus. (b, g) newly formed lenticular GC attached to the pollen wall with labelled compact GC nucleus and larger diffuse VC nucleus. The VC nucleus is smooth and round in profile at various bicellular pollen stages (g-r), irregular in early tricellular (s) stage and strongly lobed in late tricellular (t) pollen. Scale bar = 5 µm.


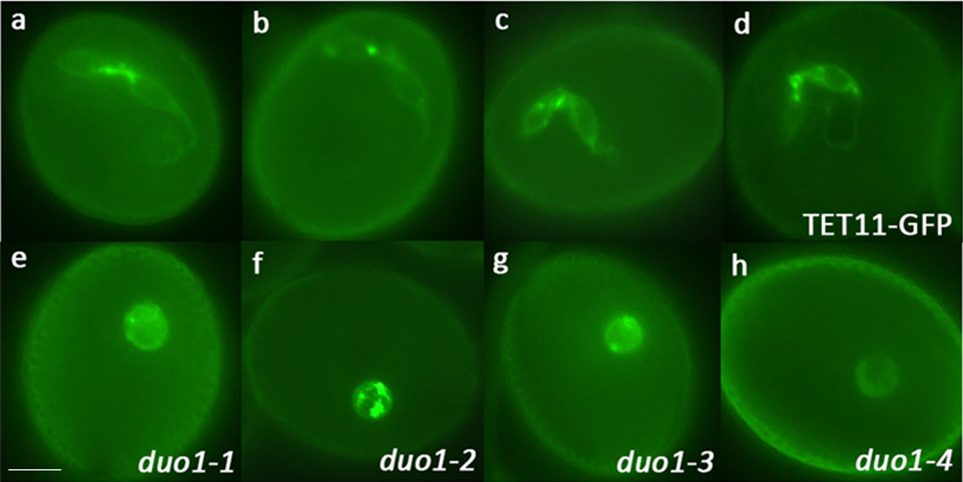


**Fig. S5.** Lack of generative cell elongation in pollen of multiple *duo1* alleles. Corresponding images of segregating normal tricellular pollen (a-d) and abnormal pollen (e-h) of heterozygous allelic *duo1* mutants marked with TET11:GFP. The GC present in abnormal (*duo1*) pollen fails to elongate. Scale bar = 5 µm.

**Fig. S6.**Analysis of pollen categories in wild type plants and in heterozygous *duo1* mutants at -4 stage flower buds scored using DIC microscopy. For each mutant, the GC in half of the pollen population had progressed to late elongation stage (ED2) or divided to form two sperm cells in tricellular pollen (TCP), while the remaining half contained a round (RD) GC. The data represent the average composition of individual plants (*N* =3) for each genotype and 60 – 156 pollen grains were counted per individual. In chi-squared tests the distributions of pollen among categories in each mutant were significantly from that of WT plants (P<.001), but the proportions of round GCs in each mutant were not significantly different from each other (P=1.0).

**
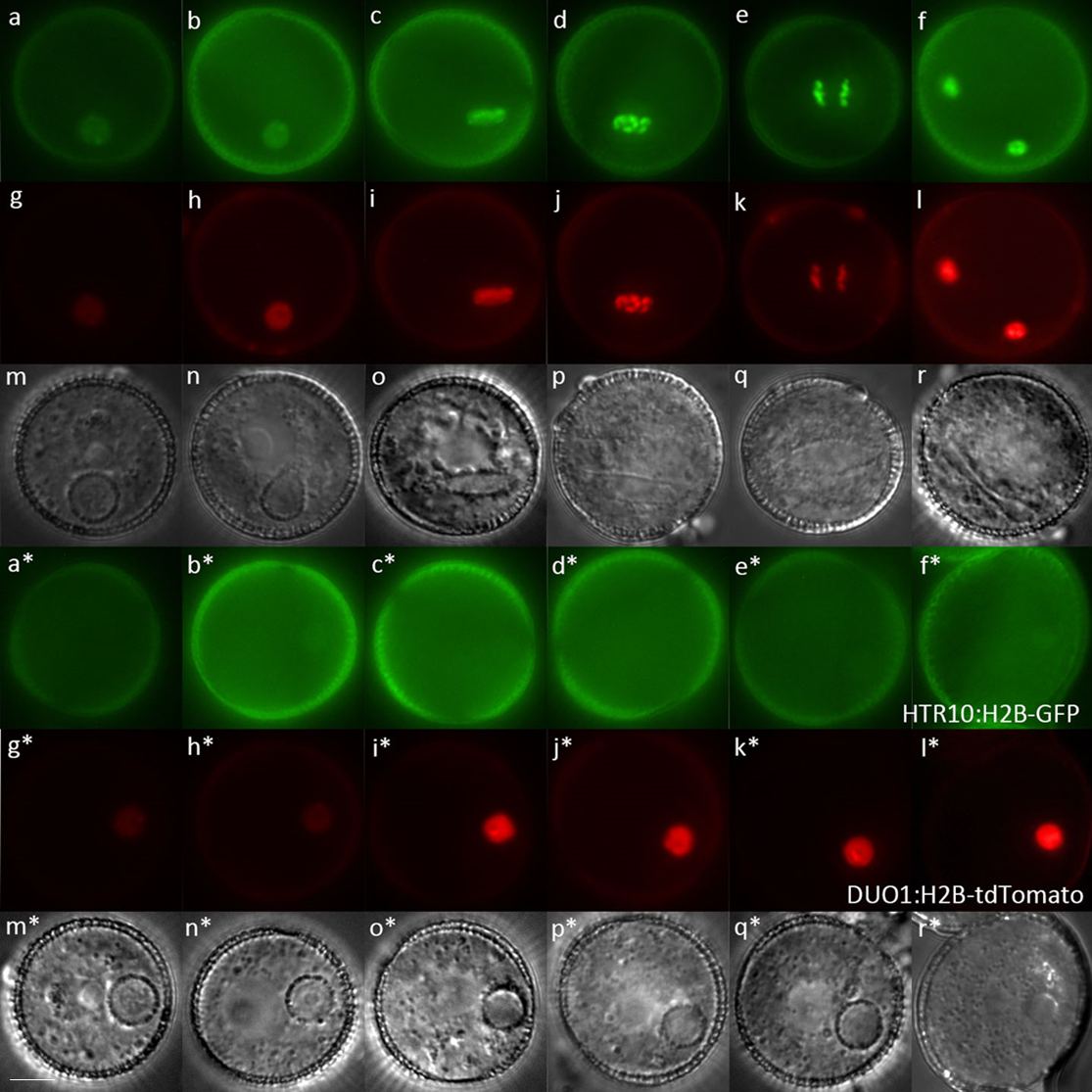
**

**Fig. S7.** Lack of generative cell elongation in *duo1-4* pollen*.* Two series of images (a-r) and (a*-r*) show double labelled (HTR10:H2B-GFP, DUO1:H2B-tdTomato) WT and mutant *duo1-4* pollen respectively at corresponding developmental stages. DUO1:H2B-tdTomato marks WT and *duo1-4* germline nuclei, while HTR10:H2B-GFP marks WT but not *duo1-4* and DIC images are shown below. The GC and the GC nucleus in *duo1-4* pollen remain round in profile (m*-r*), while both undergo elongation and divide to form two SCs in WT pollen (e-f). Scale bar = 5 µm. (a, g, m) early bicellular, (b, h, n) early bicellular, (c, I, o) late bicellular, (d, j, p) GC prophase, (e, k, q) GC anaphase, (f, l, r) GC telophase

**
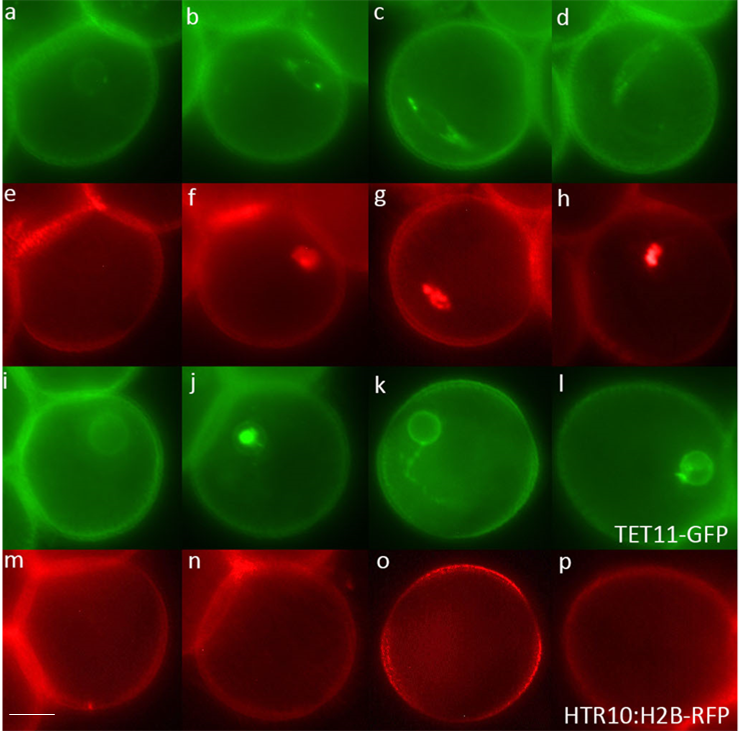
**

**Fig. S8.** Mutant *duo1-4* generative cells fail to elongate at bicellular pollen stages. Corresponding images of wild type and *duo1-4* pollen from early (a, e, I, m) to late (d, h, l, p) bicellular stages segregating in a heterozygous *duo1-4^+/-^* mutant double-labelled with TET11-GFP (a-d; i-l) and HTR10:H2B-RFP (e-h; m-p). GCs in *duo1-4* pollen which do not express HTR10:H2B-RFP (m-p), are able to form a long cytoplasmic projection (clearly visible in k), but fail to elongate (i-l), while wild type GCs show axial elongation (a-d). Scale bar = 5 µm.

**
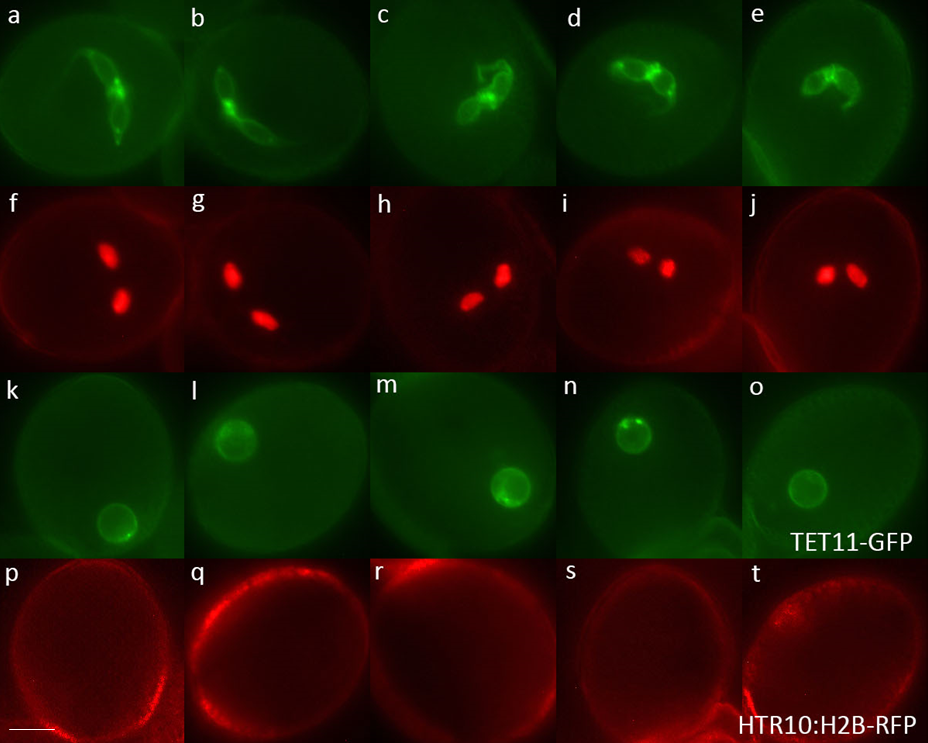
**

**Fig. S9.** Mutant *duo1-4* generative cells fail to elongate in tricellular pollen. Corresponding images of wild type and *duo1-4* pollen at early (a, f, k, p) to late (e, j, o, t) tricellular stages segregating in a heterozygous *duo1-4^+/-^* mutant double-labelled with TET11-GFP (a-e; k-o) and HTR10:H2B-RFP (f-j; p-t). GCs in *duo1-4* pollen which do not express HTR10:H2B-RFP (p-t)*,* fail to elongate and do not divide (k-o), while wild type pollen contains two elongated SCs (a-d). Scale bar = 5 µm.

**
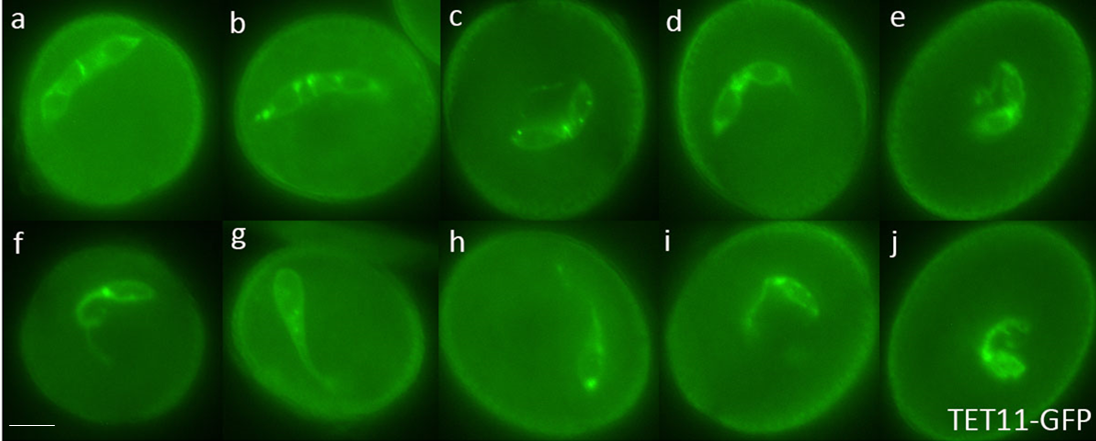
**

**Fig. S10.** Normal GC morphogenesis in *fbl17* mutant pollen*.* (a-e) Images of TET11-GFP labelled wild type pollen from late bicellular stage (a) to mature tricellular stage (e) segregating in a heterozygous *fbl17* mutant. The GC in *fbl17* pollen forms a long cytoplasmic extension and elongates normally but fails to divide (f-j), while the wild type GC divides to form two elongated SCs (a-e). Scale bar = 5 µm.

**
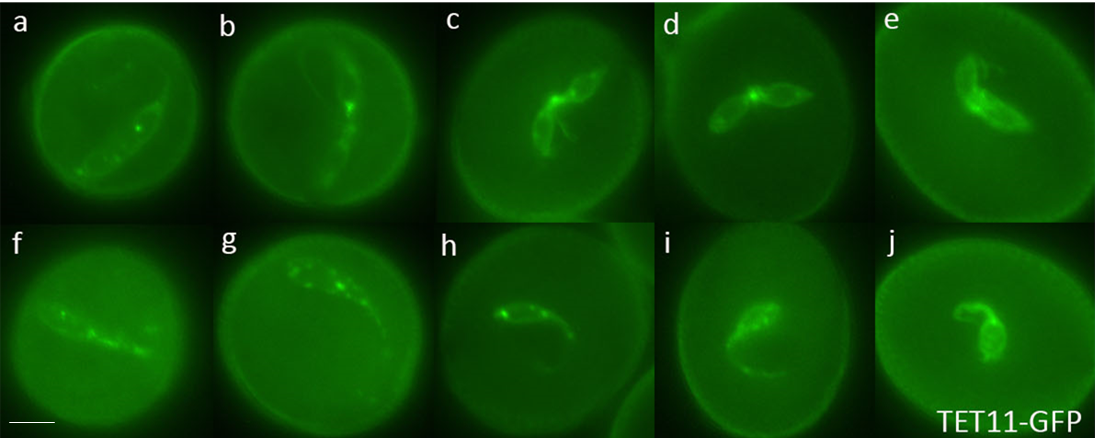
**

**Fig. S11.** Normal GC morphogenesis in *cdka;1* pollen. (a-e) Images of TET11-GFP labelled wild type pollen from late bicellular stage (a) to mature tricellular stage (e) segregating in a heterozygous *cdka;1^+/^* mutant. The GC in *cdka;1* pollen forms a long cytoplasmic extension and elongates normally but fails to divide (f-j), while the wild type GC divides to form two elongated SCs (a-e). Scale bar = 5 µm.

*
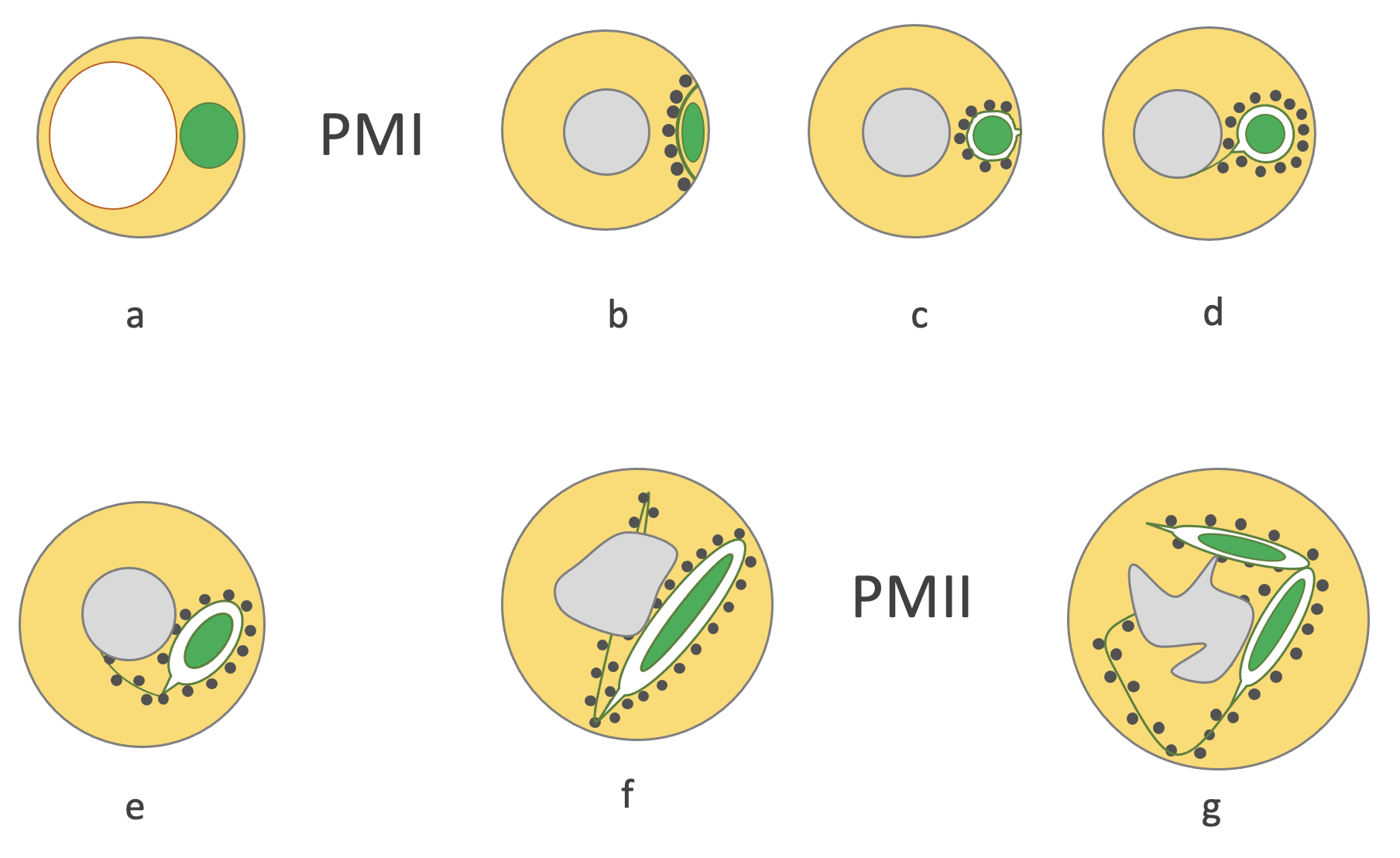
*

**Fig. S12.** Summary of angiosperm male gametophyte development and germline morphogenesis. The polarized microspore (a) divides asymmetrically at pollen mitosis I (PMI), to form early bicellular pollen (b) with a lens-shaped generative cell (GC) surrounded by a hemispherical callose wall (green line). The callose wall is then degraded and the GC rounds up before detaching from the pollen wall (c) to form a spherical GC with a fine cytoplasmic projection linked to the VC nucleus (d). The GC elongates between mid-bicellular (e), and late bicellular (f) pollen stages and the cytoplasmic extension is well developed. The elongated GC undergoes pollen mitosis II (PMII) to form two sperm cells (g), with one or both being linked to the VC nucleus by a cytoplasmic projection (g). Typically, lipid droplets in the VC cytoplasm (dots) mark the VC endo-plasma membrane surrounding the GC and cytoplasmic projection. The large microspore vacuole is outlined in red (a). Nuclei in the microspore, GC and sperm cells are shaded green. The VC nucleus is shaded grey.

**References**

Asai, Y., Takeda, K., Kamiyama, T. and Ogawa, S., (2013) Reddish-brown granules observed within the cytoplasm of generative cells of pollen grains in *Hymenocallis littoralis* (Amaryllidaceae). Cytologia, 78(3): 313-320.

Åström, H., Sorri, O. and Raudaskoski, M., (1995) Role of microtubules in the movement of the vegetative nucleus and generative cell in tobacco pollen tubes. Sexual Plant Reproduction, 8(2): 61-69.

Banaś, M., Tirlapur, U.K., Charzyńska, M., Cresti, M. (1996) Some events of mitosis and cytokinesis in the generative cell of *Ornithogalum virens* L. Planta*,* 199: 202-208.

Boavida, L.C., Qin, P., Broz, M., Becker, J.D., McCormick, S. (2013) Arabidopsis tetraspanins are confined to discrete expression domains and cell types in reproductive tissues and form homo- and heterodimers when expressed in yeast. Plant Physiology, 163: 696-712.

Borg, M., Brownfield, L. and Twell, D., (2009) Male gametophyte development: a molecular perspective. Journal of Experimental Botany, 60(5): 1465-1478.

Borg, M., Rutley, N., Kagale, S., Hamamura, Y., Gherghinoiu, M., Kumar, S., Sari, U., Esparza-Franco, M.A., Sakamoto, W., Rozwadowski, K. and Higashiyama, T., Twell, D. (2014) An EAR-dependent regulatory module promotes male germ cell division and sperm fertility in Arabidopsis. The Plant Cell, *26*(5): 2098-2113.

Brighigna, L., Fiordi, A.C., Palandri, M.R. (1981) Ultrastructural investigations on the two-nucleate pollen grain of *Tillandsia caput-medusae* Morr. (Bromeliaceae). American Journal of Botany: 1033-1041.

Brownfield, L., Hafidh, S., Durbarry, A., Khatab, H., Sidorova, A., Doerner, P., Twell, D. (2009) Arabidopsis DUO POLLEN3 is a key regulator of male germline development and embryogenesis. The Plant Cell*,* 21: 1940-1956.

Burgess, J., (1970) Cell shape and mitotic spindle formation in the generative cell of *Endymion non-scriptus*. Planta, 95(1), 72-85.

Cai, G., Del Casino, C., Romagnoli, S. and Cresti, M., (2005) Pollen cytoskeleton during germination and tube growth. Current Science, 1853-1860.

Caiola, M.G., Banas, M. and Canini, A., (1993) Ultrastructure and germination percentage of *Crocus biflorus* Miller subsp. *biflorus* (Iridaceae) pollen. Botanica Acta, 106(6), 488-495.

Charzyńska, M., Ciampolini, F. and Cresti, M., (1988) Generative cell division and sperm cell formation in barley. Sexual Plant Reproduction, *1*(4), 240-247.

Cresti, M. and Ciampolini, F., (1999) Ultrastructural characteristics of pollen development in *Vitis vinifera* L. (cv. Sangiovese). VITIS-GEILWEILERHOF*-*, 38(4), 141-144.

Cresti, M., Lancelle, S.A. and Hepler, P.K., (1987) Structure of the generative cell wall complex after freeze substitution in pollen tubes of *Nicotiana* and *Impatiens*. Journal of Cell Science, 88(3), 373-378.

Cresti, M., Milanesi, C., Salvatici, P. and Van Aelst, A.C., (1990) Ultrastructural observations of *Papaver rhoeas* mature pollen grains. Botanica Acta, 103(4), 349-354.

Cresti, M., Milanesi, C., Tiezzi, A., Ciampolini, F. and Moscatelli, A., (1988) Ultrastructure of *Linaria vulgaris* pollen grains. Acta Botanica Neerlandica, 37(3): 379-386.

Del Casino, C., Bohdanowicz, J., Lewandowska, B., Cresti, M. (1999) The organization of microtubules during generative-cell division in *Convallaria majalis.* Protoplasma*,* 207: 147-153.

Del Casino, C., Tiezzi, A., Wagner, V.T. and Cresti, M., (1992) The organization of the cytoskeleton in the generative cell and sperms of *Hyacinthus orientalis.* Protoplasma, 168(1): 41-50.

Dubas, E., Wedzony, M., Custers, J., Kieft, H., van Lammeren, A.A. (2012) Gametophytic development of *Brassica napus* pollen in vitro enables examination of cytoskeleton and nuclear movements. Protoplasma*,* 249: 369-377.

Durbarry, A., Vizir, I. and Twell, D., (2005) Male germ line development in Arabidopsis. duo pollen mutants reveal gametophytic regulators of generative cell cycle progression. Plant Physiology, 137(1): 297-307.

Ekici, N. and Dane, F., (2012) Some histochemical features of anther wall of *Leucojum aestivum* (Amaryllidaceae) during pollen development. Biologia, 67(5): 857-866.

Eliseu, S.A. and Dinis, A.M., (2008) Ultrastructure and cytochemistry of *Eucalyptus globulus* (Myrtaceae) pollen grain. Grana, 47(1): 39-51.

Eva, M., Papini, A., Brighigna, L. (2006) Ultrastructural studies on bicellular pollen grains of *Tillandsia seleriana* Mez (Bromeliaceae), a neotropical epiphyte. Caryologia*,* 59: 88-97.

Heslop-Harrison, J., Heslop-Harrison, J. S., Heslop-Harrison, Y. (1986) The comportment of the vegetative nucleus and generative cell in the pollen and pollen tubes of *Helleborus foetidus* L. Annals of Botany, 58: 1-12.

Hirano, T., Hoshino, Y. (2010) Sperm dimorphism in terms of nuclear shape and microtubule accumulation in *Cyrtanthus mackenii*. Sexual Plant Reproduction*,* 23: 153-162.

Hu, S.Y. and Yu, H.S., (1988) Preliminary observations on the formation of the male germ unit in pollen tubes of *Cyphomandra betacea* sendt. Protoplasma, 147(1): 55-63.

Iwakawa, H., Shinmyo, A., Sekine, M. (2006) Arabidopsis CDKA; 1, a cdc2 homologue, controls proliferation of generative cells in male gametogenesis. The Plant Journal, 45: 819-831.

Jensen, W.A., Fisher, D.B. (1968) Cotton embryogenesis: the sperm. Protoplasma*,* 65: 277-286.

Kartal, C. (2015). Microsporogenesis, microgametogenesis and in vitro pollen germination in the endangered species *Fritillaria stribrnyi* (Liliaceae). Caryologia, 68(1): 36-43.

Khatab, H.A. (2012) Molecular and genetic mechanisms regulating sperm cell development in *Arabidopsis thaliana*. PhD Thesis, University of Leicester.

Kim, H.J., Oh, S.A., Brownfield, L., Hong, S.H., Ryu, H., Hwang, I., Twell, D. and Nam, H.G., (2008) Control of plant germline proliferation by SCFFBL17 degradation of cell cycle inhibitors. Nature, 455(7216): 1134-1137.

Kliwer, I., Dresselhaus, T. (2010) Establishment of the male germline and sperm cell movement during pollen germination and tube growth in maize. Plant Signaling & Behavior, 5: 885-889.

Kozłowska, M., Niedojadło, K., Brzostek, M., & Bednarska-Kozakiewicz, E. (2016) Epigenetic marks in the Hyacinthus orientalis L. mature pollen grain and during in vitro pollen tube growth. Plant Reproduction, 29(3): 251-263.

Laitiainen, E., Nieminen, K.M., Vihinen, H. and Raudaskoski, M., (2002) Movement of generative cell and vegetative nucleus in tobacco pollen tubes is dependent on microtubule cytoskeleton but independent of the synthesis of callose plugs. Sexual Plant Reproduction, 15(4): 195-204.

Lancelle, S.A., Cresti, M. and Hepler, P.K., (1987) Ultrastructure of the cytoskeleton in freeze-substituted pollen tubes of *Nicotiana alata*. Protoplasma, 140(2) :141-150.

Lattar, E.C., Galati, B.G. and Ferrucci, M.S., (2012) Ultrastructural study of pollen and anther development in *Luehea divaricata* (Malvaceae, Grewioideae) and its systematic implications: Role of tapetal transfer cells, orbicules and male germ unit. Flora-Morphology, Distribution, Functional Ecology of Plants, 207(12): 888-894.

Luegmayr, E. (1993) Pollen of Hawaiian Cyrtandra (Gesneriaceae). Blumea, 38: 25-38.

McCoy, K. and Knox, R.B., (1988) The plasma membrane and generative cell organization in pollen of the mimosoid legume, *Acacia retinodes*. Protoplasma, 143(2): 85-92.

Mogensen, H. (1986) Juxtaposition of the generative cell and vegetative nucleus in the mature pollen grain of amaryllis (*Hippeastrum vitatum*). Protoplasma*,* 134: 67-72.

Murgia, M. and Wilms, H.J., (1987) Ultrastructure of the tricellular pollen grains of diploid *Euphorbia dulcis*. Caryologia, 40(3): 207-220.

Murgia, M., Detchepare, S., Van Went, J.L. and Cresti, M., (1991) *Brassica napus* pollen development during generative cell and sperm cell formation. Sexual Plant Reproduction, 4(3): 176-181.

Murgia, M., Wilms, H., Cresti, M., Cesca, G. (1986) Ultrastructure of pollen development in *Euphorbia dulcis* L. 1. Diploid plants. Acta Botanica Neerlandica, 35: 405-424.

Niu, J., Bai, H., Li, L., Zhang, X. (1999) Dynamic Association between the Vegetative Nucleus and the Generative Cell in *Tropaeolum majus* (Tropaeolaceae). Cytologia*,* 64: 215-221.

Noguchi, T. and Ueda, K., (1990) Structure of pollen grains of *Tradescantia reflexa* with special reference to the generative cell and the ER around it. Cell Structure and Function, 15(6): 379-384.

Nowack, M.K., Grini, P.E., Jakoby, M.J., Lafos, M., Koncz, C. and Schnittger, A., (2006) A positive signal from the fertilization of the egg cell sets off endosperm proliferation in angiosperm embryogenesis. Nature Genetics, *38*(1): 63-67.

Oh, S. A., Pal, M. D., Park, S. K., Johnson, J. A., Twell, D. (2010) The tobacco MAP215/Dis1-family protein TMBP200 is required for the functional organization of microtubule arrays during male germline establishment. Journal of Experimental Botany, 61: 969-981.

Ôta, T. (1957) Division of the generative cell in the pollen tube. Cytologia*,* 22: 15-27

Palevitz, B. (1993) Relationship between the generative cell and vegetative nucleus in pollen tubes of *Nicotiana tabacum*. Sexual Plant Reproduction*,* 6: 1-10.

Palevitz, B., Cresti, M. (1988) Microtubule organization in the sperm of *Tradescantia virginiana*. Protoplasma*,* 146: 28-34.

Palevitz, B.A. and Cresti, M., (1989) Cytoskeletal changes during generative cell division and sperm formation in *Tradescantia virginiana*. Protoplasma, 150(1): 54-71.

Palevitz, B.A. and Liu, B., (1992) Microfilaments (F-actin) in generative cells and sperm: an evaluation. Sexual Plant Reproduction, 5(2): 89-100.

Palevitz, B.A., Liu, B. and Joshi, H.C., (1994) γ-tubulin in tobacco pollen tubes: association with generative cell and vegetative microtubules. Sexual Plant Reproduction, 7(4): 209-214.

Rose, A., Meier, I. (2001) A domain unique to plant RanGAP is responsible for its targeting to the plant nuclear rim. Proceedings of the National Academy of Sciences of the United States of America, 98: 15377-15382.

Rotman, N., Durbarry, A., Wardle, A., Yang, W.C., Chaboud, A., Faure, J., Berger, F., Twell, D. (2005) A novel class of MYB factors controls sperm-cell formation in plants. Current Biology*,* 15: 244-248.

Rougier, M., Jnoud, N., Said, C., Russell, S. and Dumas, C., (1991) Male gametophyte development and formation of the male germ unit in *Populus deltoides* following compatible pollination. Protoplasma, 162(2): 140-150.

Russell, S.D., Strout, G.W. (2005) Microgametogenesis in *Plumbago zeylanica* (Plumbaginaceae). 2. Quantitative cell and organelle dynamics of the male reproductive cell lineage. Sexual Plant Reproduction, 18: 113-130.

Saito, C., Ueda, T., Abe, H., Wada, Y., Kuroiwa, T., Hisada, A., Nakano, A. (2002) A complex and mobile structure forms a distinct sub region within the continuous vacuolar membrane in young cotyledons of Arabidopsis. The Plant Journal, 29: 245-255.

Sanger, J.M., Jackson, W.T. (1971) Fine structure study of pollen development in *Haemanthus katherinae* Baker. II. Microtubules and elongation of the generative cells. Journal of Cell Science, 8: 303-315.

Schröder, M.B., (1985) Ultrastructural studies on plastids of generative and vegetative cells in Liliaceae 3. Plastid distribution during the pollen development in *Gasteria verrucosa* (Mill.) Duval. Protoplasma, 124(1): 123-129.

Schröder, M.B., (1986) Ultrastructural studies on plastids of generative and vegetative cells in Liliaceae. Theoretical and Applied Genetics, 72(6): 840-844.

Shi, L., Mogensen, H.L., Zhu, T. and Smith, S.E., (1991) Dynamics of nuclear pore density and distribution patterns within developing pollen: implications for a functional relationship between the vegetative nucleus and the generative cell. Journal of Cell Science, 99(1): 115-120.

Takahashi, M., (1987) Development of omniaperturate pollen in *Trillium kamtschaticum* (Liliaceae). American Journal of Botany, 74(12): 1842-1852.

Tanaka, I. (1988) Isolation of generative cells and their protoplasts from pollen of *Lilium longiflorum*. Protoplasma, 142: 68-73.

Tanaka, I. and Ito, M., (1981) Studies on microspore development in liliaceous plants III. Pollen tube development in lily pollens cultured from the uninucleate microspore stage. Plant and Cell Physiology, 22(1): 149-153.

Theunis, C., McConchie, C., Knox, R. (1985) Three-dimensional reconstruction of the generative cell and its wall connection in mature bicellular pollen of *Rhododendron*. Micron and Microscopica Acta, 16: 225-231.

Theunis, C.H., Pierson, E.S. and Cresti, M., (1992) The microtubule cytoskeleton and the rounding of isolated generative cells of *Nicotiana tabacum*. Sexual Plant Reproduction, 5(1): 64-71.

Ueda, K., Matsuyama, T., Hashimoto, T. (1999) Visualization of microtubules in living cells of transgenic *Arabidopsis thaliana.* Protoplasma*,* 206: 201-206.

Van der Maas, H.M., De Jong, E.R., Van Aelst, A.C., Verhoeven, H.A., Krens, F.A. and Van Went, J.L., (1994) Cytological characterization of isolated sperm cells of perennial ryegrass (*Lolium perenne* L.). Protoplasma, 178(1), pp.48-56.

Van Lammeren, A.A.M., Keijzer, C.J., Willemse, M.T.M. and Kieft, H., (1985) Structure and function of the microtubular cytoskeleton during pollen development in *Gasteria verrucosa* (Mill.) H. Duval. Planta, 165(1): 1-11.

Wagner, V.T. and Mogensen, H.L., (1988) The male germ unit in the pollen and pollen tubes of *Petunia hybrida*: ultrastructural, quantitative and three-dimensional features. Protoplasma, 143(2): 101-110.

Yu, H., Russell, S. (1992) Male cytoplasmic diminution and male germ unit in young and mature pollen of *Cymbidium goeringii*: a 3-dimensional and quantitative study. Sexual Plant Reproduction, 5: 169-181.

Yu, H., Russell, S.D. (1994) Male reproductive cell development in *Nicotiana tabacum*: male germ unit associations and quantitative cytology during sperm maturation. Sexual Plant Reproduction, 7: 324-332.

Zee, S.Y., (1991) Mitosis and microtubule organizational changes in isolated generative cells of *Allemanda neriifolia*. Sexual Plant Reproduction, 4(2): 132-137.

Zee, S.Y., (1992) Ultrastructure and microtubule organization of isolated generative cells of *Allemanda neriifolia* at interphase and prophase. Sexual Plant Reproduction, 5(1): 27-33.

Zee, S., Ye, X. (2000) Microtubule reorganization during pollen development of rice (*Oryza sativa* L.). Protoplasma*,* 210: 188-201.

Zhang, H., Bohdanowicz, J., Pierson, E.S., Li, Y., Tiezzi, A., Cresti, M. (1995) Microtubular organization during asymmetrical division of the generative cell in *Gagea lutea.* Journal of Plant Research, 108: 269-276.

Zhou, C. and Yang, H.Y., (1991) Microtubule changes during the development of generative cells in *Hippeastrum vittatum* pollen. Sexual Plant Reproduction, 4(4): 293-297.

Zhu, T., Mogensen, H., Smith, S.E. (1990) Generative cell composition and its relation to male plastid inheritance patterns in *Medicago sativa.* Protoplasma*,* 158: 66-72.

Zienkiewicz, K., Suwińska, A., Niedojadło, K., Zienkiewicz, A. and Bednarska, E., (2011) Nuclear activity of sperm cells during *Hyacinthus orientalis* L. *in vitro* pollen tube growth. Journal of Experimental Botany, 62(3): 1255-1269.
