# Supplementary material for: Genetic control of generative cell shape by DUO1 in Arabidopsis
Source: Plant Reprod. 2023 Apr 6;36(3):243–54. doi: 10.1007/s00497-023-00462-x (PMC10363056; doi:10.1007/s00497-023-00462-x)
Supplement: Supplementary file 2 — Supplementary file2 (DOCX 12 kb) [file 497_2023_462_MOESM2_ESM.docx]

**Supplemental movie legends**

**Movie 1.** 3-D reconstruction of a wild type Arabidopsis GC in early bicellular pollen marked with HTR10:GFP-TUA6. This cytoplasmic marker reveals the spheroidal shape of the GC.

**Movie 2.** 3-D reconstruction of a wild type Arabidopsis GC in early bicellular pollen marked with TET11-GFP. The spheroidal GC shape and the single cytoplasmic projection is highlighted with this plasma membrane marker.

**Movie 3.** 3-D reconstruction of a wild type Arabidopsis GC in mid bicellular pollen marked with HTR10:GFP-TUA6. Axial elongation of the GC towards the cytoplasmic projection is highlighted by this cytoplasmic marker.

**Movie 4.** 3-D reconstruction of a wild type Arabidopsis GC in mid bicellular pollen marked with TET11-GFP. Partial elongation of the GC and the cytoplasmic projection are highlighted by this plasma membrane marker.

**Movie 5.** 3-D reconstruction of a wild type Arabidopsis GC in late bicellular pollen marked with HTR10:GFP-TUA6. The GC extends nearly the whole pollen diameter and possess a long cytoplasmic projection at one end while the other end appears blunt.

**Movie 6.** 3-D reconstruction of a wild type Arabidopsis GC at late bicellular pollen stage marked with HTR10:GFP-TUA6. The elongated GC body has cytoplasmic projections of unequal length at its ends.

**Movie 7.** 3-D reconstruction of a pair of wild type Arabidopsis SCs in early tricellular pollen marked with HTR10:GFP-TUA6. The newly formed elongated SCs possess unequal cytoplasmic projections.

**Movie 8.** 3-D reconstruction of wild type Arabidopsis SCs in tricellular pollen marked with HTR10:GFP-TUA6. The elongated SCs are of similar length but possess unequal cytoplasmic projections.

**Movie 9.** 3-D reconstruction of wild type Arabidopsis SCs at tricellular pollen stage marked with TET11-GFP. The elongated SCs are of similar length but possess unequal cytoplasmic projections.

**Movie 10.** 3-D reconstruction of a wild type Arabidopsis VC nucleus in early bicellular pollen marked with LAT52:RanGAP-GFP. The VC appears spheroidal with a generally uniform nuclear envelope profile.

**Movie 11.** 3-D reconstruction of a wild type Arabidopsis VC nucleus in tricellular pollen marked with LAT52:RanGAP-GFP. The nuclear envelope of the VC is lobed and highly folded.

**Movie 12.** 3-D reconstruction of a mutant Arabidopsis *duo1-4* GC in tricellular pollen marked with TET11-GFP. The GC body is spheroidal but possesses a long cytoplasmic projection.
